# Novel genome and genome-wide SNPs reveal early fragmentation effects in an edge-tolerant songbird population across an urbanized tropical metropolis

**DOI:** 10.1038/s41598-018-31074-5

**Published:** 2018-08-24

**Authors:** David J. X. Tan, Balaji Chattopadhyay, Kritika M. Garg, Emilie Cros, Per G. P. Ericson, Martin Irestedt, Frank E. Rheindt

**Affiliations:** 1 Department of Biological Sciences, National University of Singapore, 14 Science Drive 4, Singapore, 117543 Singapore; 2 Department of Zoology, Swedish Museum of Natural History, P.O. Box 50007, SE-104 05, Stockholm, Sweden; 3 Department of Bioinformatics and Genetics, Swedish Museum of Natural History, P.O. Box 50007, SE-104 05, Stockholm, Sweden

## Abstract

Although edge-tolerant species are known to benefit from habitat fragmentation, less is known about the population genetic impacts fragmentation may exert on edge-tolerant species. We examined the landscape genomic structure of an edge-tolerant forest-dependent bird species, the Striped Tit-Babbler *Mixornis gularis*, in the heavily urbanized island of Singapore to determine if two centuries of fragmentation have led to signs of isolation and loss of population-genetic diversity in different parts of the island. We obtained a high-quality complete reference genome with 78x coverage. Using almost 4000 SNPs from double-digest RAD-Sequencing across 46 individuals, we found that the population has likely experienced a recent contraction in effective population size and presently exhibits low population genetic diversity. Using empirical and simulation-based landscape genomic analyses, we also found that the subtle population genetic structure observed in the Striped Tit-Babbler population in Singapore is likely driven by isolation by distance resulting from limited dispersal. Our results demonstrate that population genetic impoverishment and subdivision can accumulate at relatively rapid rates in edge-tolerant bird species such as the Striped Tit-Babbler as a result of fragmentation, and that subtle spatial genetic structure can be detected over fine spatial and temporal scales using relatively few multilocus genomic SNPs.

## Introduction

Anthropogenic habitat fragmentation is a key driver of biodiversity loss worldwide^1–3^. Organisms living in fragmented landscapes experience reduced gene flow between remnant habitat patches^4^, which may result in reduced genetic diversity, increased likelihood of inbreeding depression, higher susceptibility to stochastic environmental change, and an elevated risk of localised extirpation^4^.

The time lag between physical fragmentation and the manifestation of deleterious effects and localised extirpation results in fragmented landscapes incurring an extinction debt that may only be realised after decades^3,5–8^. Sensitive species often go rapidly extinct after initial habitat loss, followed by the gradual decline of less sensitive species due to the effects of isolation and other ecological factors^9–12^. Assessing and predicting fragmentation impacts is therefore challenging as organisms respond differentially depending on their life history traits, as well as the spatial and temporal scale of fragmentation.

While edge-tolerant species are assumed to be well-adapted to habitat fragmentation – many studies show a positive correlation between the abundance of edge-tolerant species and fragmentation effects^13–16^ – few have explored the population genetic impacts of fragmentation on these ostensibly resilient species, especially at fine spatial and temporal scales (see Harrisson *et al*.^17^ for an exception). The time lag between fragmentation and the detection of its effects, compounded by the relative abundance of edge-tolerant species, may result in (1) allele frequency-based efforts using few marker loci failing to detect subtle signals of isolation and genetic divergence at shallow temporal and spatial scales and (2) edge-tolerant species being overlooked in long-term conservation plans.

In this study, we combine RAD-Sequencing^18^ with whole-genome sequencing and individual-based landscape genomic approaches^19,20^ to investigate the impact of fragmentation on the population genetic structure of an abundant edge-tolerant passerine, the Striped Tit-Babbler (*Mixornis gularis*), in Singapore. A highly social insectivore, the Striped Tit-Babbler is a widespread resident of dense scrub and secondary woodland habitats across Southeast Asia^21^. Its affinity for disturbed and degraded habitats means that the species is abundant across its range and is not considered a species of conservation concern^22^. Although little is known about the species’ breeding or dispersal ecology, it is thought that the Striped Tit-Babbler breeds cooperatively^23^, and like other babblers (family Timaliidae) is a weak disperser on account of its short wings and sedentary habits^24^, which should increase the species’ susceptibility to fragmentation. The city-state of Singapore provides an ideal landscape for investigating this owing to its history of intensive habitat fragmentation spanning approximately 200 years, with agriculture-driven fragmentation dominating for the first 150 years, followed by urbanisation-driven secondary fragmentation^25^. This has given rise to a heterogeneous landscape consisting primarily of a heavily streetscaped urban matrix with young and maturing secondary forest fragments interspersed throughout. As one of the few woodland-dependent songbird species to have maintained healthy population levels in Singapore (4,000 to 10,000 individuals, mean estimated population density of 0.94 individuals ha^−1^ forest^26^ (Fig. S19, Supplementary Information)) in spite of extensive habitat loss and fragmentation, the Striped Tit-Babbler has likely benefited from the forest edges and secondary forests created by early fragmentation. However, it is not known whether the Striped Tit-Babbler population in Singapore constitutes multiple isolated subpopulations or a single metapopulation.

**Figure 1:**
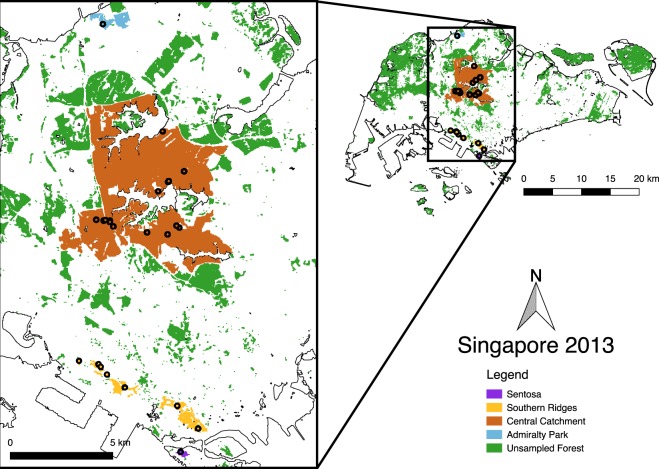
Map of Singapore with wooded areas represented in green (unsampled areas) or in additional colours other than white (sampled areas). Sampling localities of the 46 individuals are indicated as black circles. Wooded areas were classified using the maximum likelihood supervised classification method in ArcMap v10.0 based on remote sensing imagery from the LandSat OLI/TIRS platform. Subsequent map compositing was conducted in QGIS v2.18.9 (http://qgis.osgeo.org).

Using thousands of genome-wide SNPs and a newly-sequenced genome, we analyzed 46 Striped Tit-Babblers sampled from forest patches across Singapore’s north-south axis (Fig. 1) to characterize the population-genetic effects of recent secondary fragmentation in a mosaic of patches of different size and age. We used approximate Bayesian computation to assess the demographic history and effective population size (N_e_) of the Striped Tit-Babbler population in Singapore, and calculated individual and population-based divergence statistics to investigate the degree of genetic subdivision within the population. These results were compared against models of landscape structure to assess how landscape configuration affects genetic connectivity between forest fragments. To test the robustness of our SNP dataset and the informativeness of multilocus SNPs, we also assessed if varying the number of SNPs used would affect downstream population genetic inferences.

## Results

### Mist Netting

We ringed a total of 66 Striped Tit-Babblers across 19 unique sampling localities. Accounting for historical ringing records (unpublished data, National Parks Board of Singapore), 26 individuals were recaptured across 37 recapture events between the years 2010 and 2014. All recaptures occurred at the original locality of capture, suggesting a highly sedentary lifestyle. One recapture occurred eight years after the original ringing, suggesting that the species is relatively long-lived in the wild.

### Genome Assembly

After filtering out low quality and clonally duplicated reads, a total of 86 Gb of DNA (78x coverage) was obtained for *de novo* assembly of the Striped Tit-Babbler genome, and assembly quality and completeness were assessed for each assembly by checking read pair coverage and supporting evidence (Table 1). Based on standard contiguity metrics, it is clear that the ALLPATHS-LG assembly outperforms the other two assemblies, with an N50 of 3 Mb and producing 9542 scaffolds, thereby producing the fewest but longest sequences (Table 1). In addition, to evaluate the assembly correctness, we used FRCurves to plot regions of suspected mis-assemblies (features) against the coverage depth (Fig. S7, Supplementary Information). In this instance, the ALLPATHS-LG assembly also presents the best performance, with the FRCurve indicating better genome coverage with fewer suspect errors introduced relative to the ABySS and SOAPdenovo assemblies. This Whole Genome Shotgun project has been deposited at DDBJ/ENA/GenBank under the accession QVAJ00000000. The version described in this paper is version QVAJ01000000.

**Table 1:** Standard contiguity metrics for the three genome assemblies, ALLPATHS-LG, ABySS, and SOAPdenovo, showing the number of scaffolds (n.scaff), number of scaffolds longer than 1000 base pairs (n.scaff >1000), the N50 and N80 measures, the maximum scaffold length (max_scf_lgth), the total assembly length (Ass.lgth), as well as the total assembly length for scaffolds greater than 1000 base pairs (Ass_lgth_ctg > 1000).

Assembler	n.scaff	n.scaff >1000	N50	N80	max_scf_lgth	Ass.lgth	Ass_lgth_ctg >1000
ALLPATHS-LG	9,542	9,011	3,093,332	1,158,118	19,816,433	1,049,058,013	1,048,547,872
ABySS	2,003,503	65,683	88,266	23,906	1,364,009	1,252,241,058	1,006,935,606
SOAPdenovo	3,270,383	77115	75,763	33,056	2,562,666	1,913,786,802	1,237,693,708

### ddRAD-Seq Results

We obtained 386,020,580 paired-end Illumina reads of 100 base-pair length each across 47 individuals, of which 325,488,846 reads (84.3%) were retained after quality control, filtering, and trimming. The number of retained reads per individual ranged from 4,225,764 to 14,316,443, and applying further quality control and aligning these reads to the reference genome resulted in 3,017,218 to 9,898,116 reads per individual successfully mapping to the reference genome. Assembling mapped reads into loci, calling SNPs, filtering for no missing data, and filtering for linkage disequilibrium resulted in an output SNP matrix containing 3849 loci. Bayescan did not detect any loci under selection, and while some SNP loci may nonetheless be closely linked to loci with fitness effects, we assumed that all loci were neutral absent better methods for testing this. Filtering for half-sibs resulted in 11 individuals being pruned, giving a reduced SNP matrix comprised of 35 individuals. Comparing inbreeding coefficients showed that most individuals exhibit relatively low levels of inbreeding (Table S2; Fig. S9, Supplementary Information; mean TrioML coefficient = 0.0176), although individuals from the Admiralty Park subpopulation appear to be significantly more inbred (mean TrioML coefficient = 0.101), with one individual exhibiting an extreme TrioML coefficient of 0.160 (Fig. S9; Table S2, Supplementary Information).

### Population Genetic Structure and Genetic Diversity Statistics

**Figure 2:**
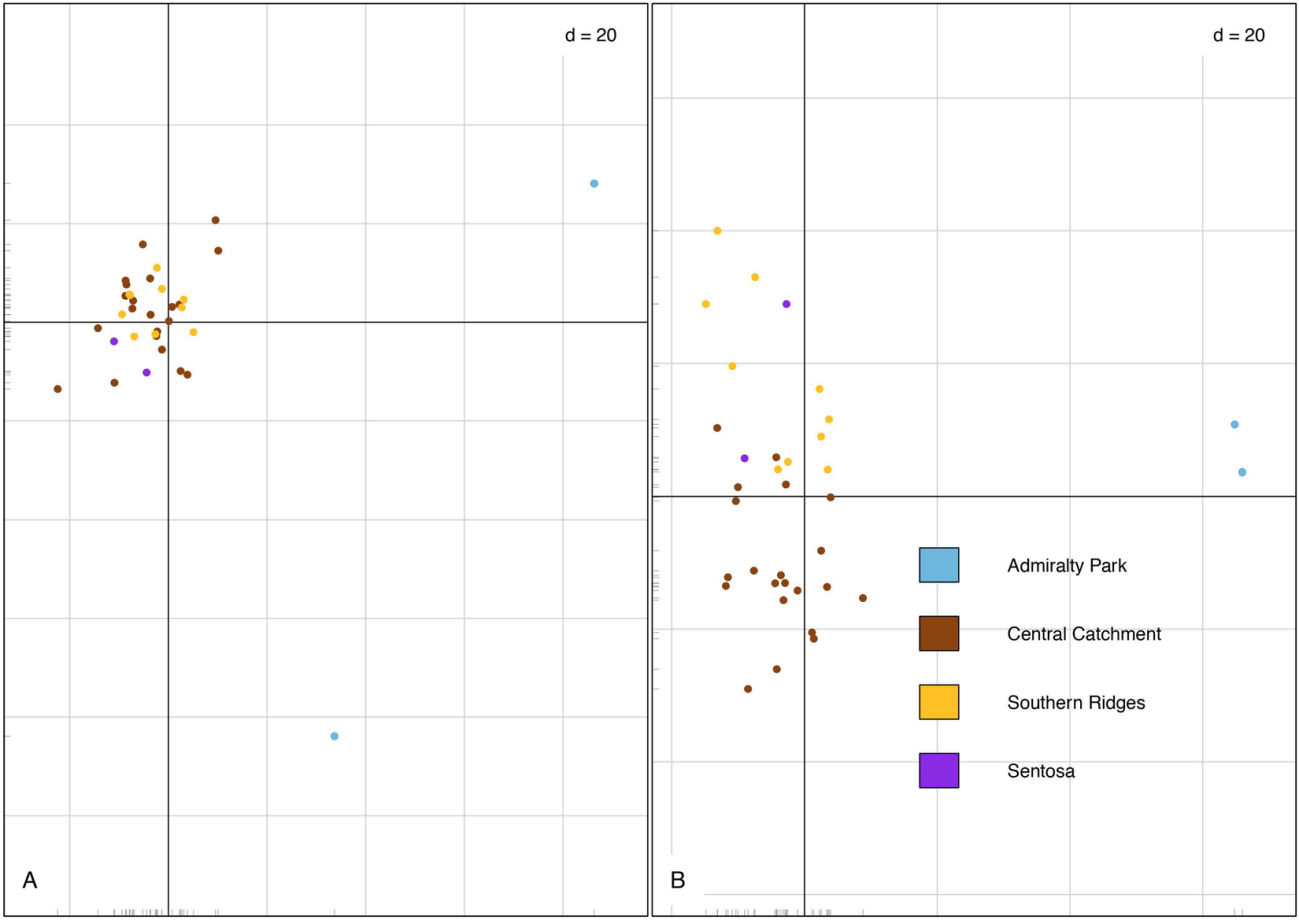
Principal components analyses of the sampled Striped Tit-Babbler individuals using (**A**) the kin filtered (n = 35) SNPset with the highly inbred individual from Admiralty Park (K1103) included, and (**B**) the kin-filtered (n = 35) SNPset with the highly inbred individual from Admiralty Park replaced with its less inbred kin (K1105). For both plots, PC1 and PC2 are plotted, with both plots showing that most of the individuals cluster into a single cloud, and the individuals from Admiralty Park falling out as outliers. The colour scheme follows that of Fig. 1.

The first three PC axes (comprising 4.00%, 3.78%, and 3.72% of the total variance, respectively) indicate that most of the Striped Tit-Babblers sampled fall into a single cluster with little to no substructure (Fig. 2). However, the PCA plot also indicates that all the individuals sampled from Admiralty Park fall out of the central cloud as outliers (Fig. 2). Based on the results of the PCA, we subsequently merged the individuals sampled from Sentosa Island with the Southern Ridges in to a single “Southern” subpopulation (Fig. 1), due to the Sentosa individuals clustering together with the individuals sampled from the Southern Ridges (Fig. 2B).

The AMOVA results show that 97.1% of the genetic variation was contained within individuals, while 2.3% of genetic variation occurred among subpopulations (F_ST_ = 0.023, p = 0.001), indicative of low levels of population genetic subdivision. Within-subpopulation inbreeding was barely statistically significant, and accounted for only 0.6% of the total genetic variation (F_IS_ = 0.006, p-value = 0.045), suggesting relatively low levels of non-random mating within subpopulations.

Using the SNP matrix containing all 46 individuals, the overall mean nucleotide diversity (π) for polymorphic loci was 0.2311, with within-subpopulation π values ranging from 0.2093 (Admiralty) to 0.2306 (Central Catchment) (Table 2). The overall observed heterozygosity value (H_obs_) was 0.2269, with within-subpopulation values ranging from 0.2096 (Admiralty) to 0.2294 (Central Catchment) (Table 2). For both fixed and polymorphic loci, π_overall_ was 0.0026, with within-subpopulation values ranging from 0.0023 (Admiralty) to 0.0026 (Central Catchment), while H_obs_ was 0.0025 with a within-subpopulation range of 0.0023 (Admiralty) to 0.0025 (Central Catchment and Southern) (Table 2). The similarity of within-subpopulation π and H_obs_ values across subpopulations suggests that Striped Tit-Babbler subpopulations in Singapore do not differ significantly from one another, although the Admiralty Park subpopulation appears to consistently exhibit the lowest levels of genetic diversity. We obtained similar π and H_obs_ values for both the kin-filtered and unfiltered analyses, suggesting that π and H_obs_ are both largely unaffected by filtering for relatedness (Table 2).

**Table 2:** Unfiltered and kin-filtered summary population statistics for both individual sub-populations and the overall population mean.

Population Cluster	Unfiltered (n = 46)					Kin-filtered (n = 35)				
	N	Private	P	H_Obs_	π	N	Private	P	H_Obs_	π
Polymorphic loci										
Southern	16	192	0.8451	0.2258	0.2271	11	213	0.8427	0.2281	0.2319
Admiralty	3	81	0.8685	0.2096	0.2093	2	77	0.8653	0.2117	0.2295
Central Catchment	27	515	0.8417	0.2294	0.2306	20	537	0.8383	0.2346	0.2367
Overall	46	0	0.8407	0.2269	0.2311	35	0	0.8376	0.2309	0.2361
Fixed & Polymorphic Loci										
Southern	16	192	0.9983	0.0025	0.0025	11	213	0.9983	0.0025	0.0026
Admiralty	3	81	0.9985	0.0023	0.0023	2	77	0.9985	0.0024	0.0026
Central Catchment	27	515	0.9982	0.0025	0.0026	20	537	0.9982	0.0026	0.0026
Overall	46	0	0.9982	0.0025	0.0026	35	0	0.9982	0.0026	0.0026

Population statistics have been split into statistics for polymorphic loci only and for both polymorphic and fixed loci. Statistics include the total average number of individuals genotyped at each locus (N), the number of alleles unique to each population (Private), average major allele frequency (P), observed heterozygosity (H_Obs_) and mean nucleotide diversity (π).

Interestingly, the mean Admiralty Park TrioML inbreeding coefficient (Fig. S9; Table S2, Supplementary Information) is consistent with H_obs_ (Table 2). Since the mean genome-wide heterozygosity of a population (H) and realised mean level of inbreeding (F) can be related in the equation H = H_0_ (1 − F), assuming the Central Catchment subpopulation to be H_0_ on account of it being the most genetically diverse subpopulation in Singapore, solving for F for both the unfiltered and kin-filtered datasets gives 0.09 and 0.10 respectively, both of which are close to the empirically derived TrioML mean inbreeding coefficient of 0.101 (Table 2; Fig. S9; Table S2, Supplementary Information).

Pairwise F_ST_ values calculated using the full (n = 46) SNP matrix ranged from 0.0124 (F_ST-CentralCatchment-Southern_, 95% CI: 0.0105–0.0144) to 0.0606 (F_ST-Southern-Admiralty_, 95% CI: 0.0525–0.0688), with relatively low pairwise F_ST_ values between the Central Catchment and the Southern subpopulation indicative of overall low population subdivision between the two largest forest patches along the north-south axis of Singapore (Table 3; Fig. S20, Supplementary Information). In contrast, moderately high (>0.05) pairwise F_ST_ values between Admiralty Park and all other forest patches suggest increased population subdivision between Admiralty Park and any other sampled forest patch. Filtering the SNP matrix for kin resulted in lower absolute F_ST_ values, ranging from 0.0073 (F_ST-CentralCatchment-Southern_, 95% CI: 0.0052–0.0094) to 0.0261 (F_ST-Southern-Admiralty_, 95% CI: 0.0169–0.0352), although relative pairwise differences between subpopulations remained largely the same (Table 3).

**Table 3:** Filtered and unfiltered Weir and Cockerham’s F_ST_ values for each pairwise population comparison, with confidence intervals (in parentheses) calculated using 9,999 bootstraps across loci.

	Southern	Admiralty	Central Catchment
Southern	—	0.0261 (0.0169,0.0352)	0.0073 (0.0052,0.0094)
Admiralty	0.0606 (0.0525, 0.0688)	—	0.0226 (0.0142,0.0310)
Central Catchment	0.0124 (0.0105,0.0144)	0.0558 (0.0483,0.0634)	—

F_ST_ values on the lower diagonal were calculated using the full unfiltered SNP matrix (n = 46) while F_ST_ values on the upper diagonal were calculated using the kin-filtered SNP matrix (n = 35).

Recalculating population genetic statistics, pairwise population differentiation and mean inbreeding coefficients using varying numbers of randomly subsampled loci, we found that the H_obs_, π, pairwise F_ST_, and TrioML inbreeding coefficients were precise for 500 or more SNP loci, even for sample sizes as small as three individuals (Figs. S10–S13, Supplementary Information).

### Analyses of Gene Flow

We identified two first-generation migrants: one individual from Sentosa island (sample K1120) within the Southern subpopulation and one from Admiralty Park (sample K1104), both of which were inferred to have originated from the Central Catchment. This pattern is indicative of recent gene flow from the Central Catchment toward peripheral subpopulations.

### Demographic History of the Striped Tit-Babbler

PCA for pre-evaluation of scenarios revealed that the observed data fall within the prior space of the contraction scenario (Scenario 2; Fig. S14). Model comparison with both rejection and logistic regression revealed overwhelming evidence of recent population decline (Fig. S15, Supplementary Information). Further posterior predictive checks using default parameters revealed that the population decline scenario has no associated error, further adding confidence to our scenario choice (Table S4, Supplementary Information). Plots of prior versus posterior revealed that for the best scenario, all posterior sampling of parameters fell within the prior space (Fig. S16, Supplementary Information). Although the confidence intervals are high, median parameter estimates suggest that the present N_e_ is three orders of magnitude lower than the ancestral N_e_ (approximately 544 effective contemporary individuals compared with 107,000 effective ancestral individuals), and that the population has suffered a recent decline approximately half a century ago (Table 4).

**Table 4:** Median, mode, and 5%, 25%, 75%, and 95 quartile estimates from DYABC posterior distribution samples of demographic parameters.

Parameter	Median	Mode	5% Quantile	25% Quantile	75% Quantile	95% Quantile
Ancestral Population Size (N_dec_)	107,000.00	691.00	1,670.00	19,300.00	367,000	828,000.00
Time of Contraction (t)	46.20	5.00	5.00	12.90	261.00	6,340.00
Current Population Size (N1)	544.00	67.70	10.00	150.00	2,930.00	30,700.00

Independently estimating N_e_ from the observed data using the linkage disequilibrium method, the full (n = 46) dataset results in a population-wide N_e_ of 101.5 (95% CIs: 100.8–102.2). Using the kin-filtered (n = 35) dataset results in a population-wide N_e_ of 596.1 (95% CIs: 569.4–625.4).

### Landscape Genetic Analyses

**Figure 3:**
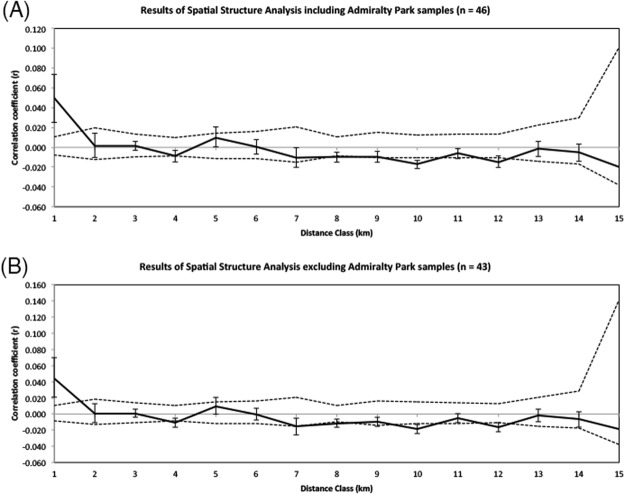
Spatial autocorrelograms showing the genetic correlation coefficient (r) for each distance bin (km) for the Striped Tit-Babbler population in Singapore. Dashed lines represent the upper and lower bounds of the 95% confidence intervals for r generated under the null hypothesis of random geographic distribution of the Striped Tit-Babbler for 999 permutations. 95% confidence interval error bars were calculated using 1,000 bootstraps over pairs of samples. Both the autocorrelograms (**A**) including the Admiralty Park individuals and (**B**) excluding the Admiralty Park individuals indicate that the Striped Tit-Babbler population in Singapore exhibits significant positive spatial autocorrelation for the first distance bin (0–1 km) and significantly negative spatial autocorrelation for the tenth and twelfth distance bins, with the high levels of genetic correlation and short distances suggesting that Striped Tit-Babbler individuals are likely to be poor dispersers.

Spatial autocorrelation analysis indicated that the Striped Tit-Babbler exhibits significantly positive spatial autocorrelation for the first distance class (0–1 km) and significantly negative spatial autocorrelation for the fourth, and the seventh to twelfth distance classes (Fig. 3), consistent with observational inferences about the poor dispersal ability of the Striped Tit-Babbler^24^. Spatial autocorrelation in the second and third distance classes did not differ significantly from zero (Fig. 3). The spatial autocorrelation analysis remained largely invariant to the exclusion of the Admiralty Park individuals (Fig. 3B).

Optimising landscape parameters showed that the optimised model with urban resistance set to 90 and managed vegetation resistance set at 50 (R^2^_adj_ = 0.0343) outperforms the preliminary model, with resistance values of 60 and 40 respectively (R^2^_adj_ = 0.0231). The proportion of genetic variation explained by spatial factors for the optimised resistance model (R^2^_adj_ = 0.0344, p = 0.000001) was higher than the Euclidean model (R^2^_adj_ = 0.0275, p = 0.000001) and preliminary resistance model (R^2^_adj_ = 0.0331, p = 0.000001), indicating that the optimised landscape resistance model provides a marginally better explanation for the spatial genetic signal observed in the Striped Tit-Babblers (Table S8, Supplementary Information). While this suggests that an IBD + IBR model best explains the landscape genetic structure of Striped Tit-Babblers, the model is closely correlated with the null IBD model (R^2^ = 0.8009, P = 0.0001), which may inflate the likelihood of type I error.

### Landscape Genetic Simulations

**Figure 4:**
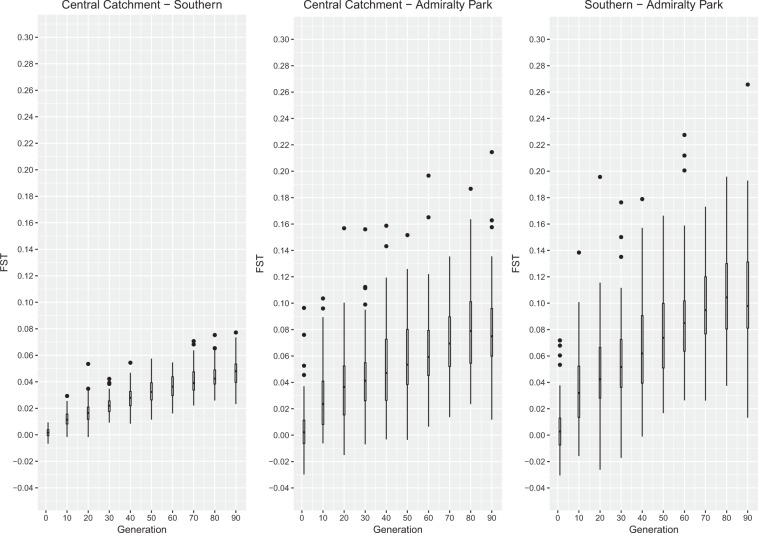
Simulated change in pairwise F_ST_ over time based on forward-in-time landscape genetic simulations, showing a relatively higher F_ST_ accumulation rate between Admiralty Park and all other subpopulations and a lower F_ST_ accumulation rate between the Central Catchment and Southern subpopulations.

We discarded seven of 100 replicate MCMC landscape genetic simulations due to stochastic extinctions of the Sentosa individuals. We observed an overall increase in F_ST_ for all pairwise subpopulation comparisons over time (Fig. 4), although the Admiralty Park subpopulation exhibits a higher accumulation rate of pairwise differentiation (Fig. 4).

Testing the CDPOP simulations for Type I errors, we detected false positive signals of IBD + IBR in 73.9% of the simulated IBD-only genotypes at generation 10, as well as in 93.97% of the simulated genotypes at generation 20, 98.9% of the simulated genotypes at generation 30, 95.6% of the simulated genotypes at generation 40, and 97.8% of the simulated genotypes at generation 50.

## Discussion

Based on the first genome assembly of the Striped Tit-Babbler *Mixornis gularis*, combined with population-genomic analyses and landscape genetic simulations from 46 individuals across Singapore, we detected a pattern of reduced N_e_ and subtle but noticeable IBD-driven population subdivision along Singapore’s North-South axis. That these patterns were detected at fine spatial and temporal scales illustrates the utility of genome-wide multilocus SNPs in illuminating the genetic impacts of habitat fragmentation prior to the manifestation of deleterious physical effects.

Coalescent simulations strongly indicate that the Striped Tit-Babbler population has experienced a recent contraction in N_e_ across Singapore, with median parameter estimates suggesting a decline by three orders of magnitude approximately 46 years ago. While there is uncertainty over the precision of these estimates, the estimated time of population contraction coincides with a period of intensive urbanisation in Singapore, and the reduction in habitat extent during this period was likely a major contributor to the contraction in N_e_.

The population-wide N_e_ estimate of ~596 derived from the kin-filtered dataset using the linkage-disequilibrium method is consistent with the DIYABC-derived contemporary population-wide median N_e_ estimate of 544. The results indicate that contemporary N_e_, and by extension overall genetic variability, is low relative to the census population size, with a conservatively estimated N_e_/N ratio of 0.15 (assuming N_e_ = 596, and census population size = 4,000 (Fig. S19, Supplementary Information)^26^. Although the N_e_/N ratio is expected to be low in birds due to unequal sex ratios^27^, especially in cooperative breeders, the Striped Tit-Babbler population in Singapore nonetheless exhibits an N_e_/N ratio 50% less than that observed in other similarly sedentary and cooperatively breeding birds such as the Splendid Fairy-wren (*Malurus splendens*) (N_e_/N = 0.3)^28^ and Darwin’s Medium Ground Finch (*Geospiza fortis*) (N_e_/N = 0.31)^27,29^, suggesting that the Striped Tit-Babbler population is likely more susceptible to the impacts of fragmentation than other species with similar life histories.

AMOVA and principal component analyses (Fig. 2) indicate that most of the sampled individuals fall into a single cluster with little variation between individuals, pointing to the presence of at least modest levels of recent gene flow (as inferred from the presence of first-generation migrants) and an overall shallow population genetic structure^30,31^. However, significant pairwise F_ST_ values between subpopulations and reduced H_obs_ and π values in the Admiralty Park subpopulation points toward the existence of some population genetic subdivision between the forest patches along the north-south axis of Singapore.

PCA results (Fig. 2) show that the individuals sampled from Admiralty Park emerge as outliers despite strict filtering conditions to account for low coverage loci, kinship bias, linkage disequilibrium, and loci under selection. Additionally, significantly higher overall TrioML inbreeding coefficients of the Admiralty Park samples (Fig. S9, Supplementary Information), consistent with H_obs_, as well as the presence of one individual exhibiting an inbreeding coefficient one order of magnitude higher than the mean Singapore-wide inbreeding coefficient (Table S2, Supplementary Information), provide evidence of a subpopulation in relative isolation.

We consider it unlikely that the signal of population subdivision observed in Admiralty Park may be an artifact of low sampling coverage or biased by PCR clones^32,33^: our study employs thousands of marker loci, and datasets of this magnitude have been shown to produce accurate F_ST_ estimates with sample sizes as low as two individuals^34^; furthermore, we sampled approximately 4–6% of the total Admiralty Park subpopulation^26^ (Fig. S19, Supplementary Information), and conducted triplicate PCR reactions to minimise the biasing effect of PCR clones.

In addition, Admiralty Park individuals consistently emerge as outliers on the PCA plot even when the highly inbred individual is replaced with its less inbred kin (sample K1105; Table S2, Supplementary Information) for the same set of SNP loci (Fig. 2B), strengthening confidence in our data.

The low genetic diversity and comparatively high subdivision of Admiralty Park from the Central Catchment is surprising owing to their close proximity (minimum Euclidean distance of approximately 2.25 km) and the relatively recent isolation of Admiralty Park (approximately 20–40 years based on satellite imagery; Fig. S18, Supplementary Information). The heterogeneity of population genetic structure observed in the Striped Tit-Babbler population in Singapore indicates that spatial context plays a significant role in affecting inter-patch gene flow.

We find that the Striped Tit-Babbler population exhibits significant positive spatial autocorrelation over short distances (<1 km), even after the potentially aberrant Admiralty Park individuals are excluded (Fig. 3B). While empirical data on natal dispersal distances of tit-babblers are limited, this result is similar to that of the relatively closely related Abbott’s Wren Babbler (*Turdinus abbotti*)^35^ obtained from radio telemetry methods (200–700 m)^36^. Limited dispersal and highly sedentary behaviour in this species (inferred from recapture data) likely contributes to the statistically significant signal of IBD observed in the dbMEM analysis. Simulating the effects of IBD using forward-in-time landscape genetic simulations further shows that a pattern of relatively stronger population differentiation from the Central Catchment emerges in the Admiralty Park subpopulation within relatively few generations compared to the Southern subpopulation (Fig. 4), consistent with empirically observed population genetic structure (Table 3). Our landscape genetic analyses strongly suggest that IBD is a primary driver of subtle population genetic structure in the Striped Tit-Babbler in Singapore.

In Admiralty Park, limited dispersal (Fig. 3), small local N_e_ (estimated at 10 effectively breeding individuals based on a conservative N_e_/N ratio of 0.15), and the paucity of forest patches within dispersing distance of other proximate source forests (Fig. 1) have likely resulted in the rapid accumulation of population subdivision over relatively few generations. In contrast, the weaker population genetic structure observed in the south may be attributable to the existence of “stepping stone” habitat patches (Fig. 1).

As for the IBR signal observed in the dbMEM analysis, the high proportion of IBD-only landscape genetic simulations showing false positive signals of IBD + IBR suggests that the sampling strategy adopted in this study lacks sufficient power for discriminating between IBD and IBR, although IBR may still apply in our study system. This outcome highlights the importance of rigorous sampling design in landscape genetic studies^37^.

Our observation that a minimum of 500 to 1,000 loci are needed for precise estimates of most population genetic statistics (Figs S10–S13, Supplementary Information) suggests that relatively few loci are needed to derive precise estimates of population genetic statistics. This is consistent with the findings of Kardos *et al*.^38^ and Nazareno *et al*.^34^, who find that using large numbers of loci does not necessarily improve the statistical power of population genetic analyses^34,38^.

While the Striped Tit-Babbler population in Singapore is not presently threatened with extirpation^39^, an examination of genome-wide SNPs reveals that the species has experienced recent decline in N_e_, has relatively low population-genetic diversity, and exhibits weak population genetic structure consistent with the effects of limited dispersal, suggesting that the population is susceptible to the effects of habitat fragmentation. Detecting these population-genetic signals in an abundant and edge-tolerant species at such fine spatial and temporal scales likely reflects the initial stages of fragmentation usually detected only in more sensitive species or at later stages in the local extirpation process. Other relatively edge-tolerant and abundant forest-dwelling species, avian or otherwise, may likewise be shown to experience such fragmentation effects once genome-wide data are applied. Our results are especially significant in the context of Southeast Asian biodiversity conservation owing to the rate at which forest habitats are being degraded and fragmented across the region^40^.

Our study on Striped Tit-Babblers in Singapore applies ddRAD-Seq and individual-based landscape genomic techniques to elucidate spatial genetic structure in an overlooked edge-tolerant forest-dwelling avian species across heavily fragmented tropical forest patches. We demonstrate that extremely fine-scale population genetic structure on the order of 2.3% of molecular variance can be detected using thousands of genome-wide marker loci. We hope that further investigations in this vein will lead to a better understanding of the fine-scale spatial effects of fragmentation and hopefully lead to solutions to ameliorate these impacts.

## Methods

### DNA Sampling

We conducted mist netting at forest fragments across Singapore between May 2013 and September 2014 (Fig. 1). We collected blood samples via brachial venipuncture, subsequently stored at 4 °C. In addition, all mist-netted birds were uniquely ringed for recapture studies.

Additional DNA samples were obtained from muscle and liver tissues stored at the Lee Kong Chian Natural History Museum and from the carcass collection of the NUS Avian Evolution Laboratory. A total of six tissue samples were obtained, five from the cryogenic collection dating to October 2006 and one from the carcass collection from March 2014.

### RAD-Seq Library Preparation

We extracted DNA using the Exgene Clinic SV kit (GeneAll Biotechnology) per the manufacturer’s protocol for blood and body fluid DNA extraction, with minor modifications for samples stored in 100% ethanol. For muscle and liver tissue samples, we extracted DNA as per the Animal Tissue protocol for the Exgene Clinic SV kit. Extracted DNA samples were eluted into molecular-grade water and stored at −20 °C.

We prepared double digest RAD-Seq libraries for each sample based on a modified FASSST protocol developed by Tay *et al*.^41^ and Tin *et al*.^33^, using combinatorial barcodes derived from Peterson *et al*.^18^ and the restriction enzymes EcoRI (NEB) and MspI (NEB). We conducted triplicate PCR reactions per sample to reduce the likelihood of PCR bias highlighted by Tin *et al*.^33^ and to maximise the yield of adapter-ligated fragments. We produced 47 successful double digest RAD-Seq libraries, inclusive of one replicate specimen, pooled in equimolar volumes. Pooled libraries were sequenced on one Illumina HiSeq2000 lane at BGI Shenzhen, producing 100 bp paired-end reads.

### Whole Genome Sequencing and Assembly

Genomic DNA was extracted from fresh tissue from one Striped Tit-Babbler individual using the KingFisher™ Duo extraction robot (Prime Magnetic Particle Processor) and the KingFisher Cell and Tissue DNA Kit, following the manufacturer’s protocol. Preparation of libraries, sequencing and the assembly of the *de novo* genome were performed by Science for Life Laboratory (SciLifeLab) in Stockholm. Short-insert-sized (180 bp) and mate-pair (5 and 8 kb) DNA libraries were constructed. All libraries were sequenced on the Illumina HiSeq2500 platform with a 2 × 126 setup in RapidHighOutput mode. Paired-end sequence data from the genomic DNA libraries were quality-checked, assembled, and evaluated using the NouGAT pipeline^42^.

### ddRAD-Seq Read Processing and Alignment

We analysed raw sequence reads with FastQC to determine average quality scores across all reads. We used the *process_radtags* pipeline in Stacks v1.3^43,44^ to demultiplex sequence reads, filter out low quality reads, and trim raw sequence reads to 90 bp. Single nucleotide errors within the barcode were corrected by the software. We used Bowtie2 v2.2.5^45^ to align the RAD reads to the Striped Tit-Babbler reference genome. Successfully aligned reads were processed with SAMtools v1.0^46^ to filter out reads with mapping quality score <25, exclude improperly paired reads, and convert the files to BAM format.

We used the *ref_map.pl* pipeline in Stacks v1.3 to assemble reference-aligned reads into loci for SNP calling, ensuring that only loci with no missing data were reported in the output SNP matrix. We used default parameters in Bayescan^47^ to detect loci under selection, and used PLINK v1.9^48^ to identify loci in linkage disequilibrium for subsequent filtering.

We used Coancestry^49^ to determine the relatedness coefficients between individuals as well as the inbreeding coefficients for all 46 individuals. We filtered out one individual from each pair with an inferred relatedness greater than half-sibs (Queller and Goodnight)^50^ relatedness coefficient >0.25) using GenoDive^51^. We used the TrioML method^52^ to estimate inbreeding coefficients as it performs better than other estimators, especially for populations with high inbreeding and closely related individuals^52,53^.

### Analysis of Population Genetic Structure

To explore inter-individual differentiation, we performed a PCA with the kin-filtered SNP matrix using the dudi.pca function in the ade4 package^54^ in R v3.2.2 (R Core Development Team, 2015). The results of the PCA were plotted using the dudi.plot function in the R package Momocs^55^.

To calculate genetic diversity statistics, we grouped the 46 samples into three putative subpopulations based on the PCA results (Fig. 2), comprising one isolated forest patch (Admiralty Park: n = 3), and two large but fragmented networks of forest patches (Southern: n = 16, and Central Catchment Nature Reserve: n = 27) (Table 2). We conducted an analysis of molecular variance (AMOVA) in GenoDive, and calculated population genetic statistics for both the full (n = 46) and kin-filtered (n = 35) datasets using the *populations* module in Stacks. We calculated pairwise Weir and Cockerham’s F_ST_^56^ using the R package diveRsity. As the removal of kin may improve the performance of F_ST_ estimations at the expense of precision^57^, we calculated pairwise F_ST_ values using both the full (n = 46) SNP matrix and the kin-filtered (n = 35) SNP matrix.

To test the robustness of our population genetic inferences, we used custom bash scripts to randomly subset the original SNP matrix for 2 to 3,500 loci, and recalculated population genetic statistics based on these reduced-loci SNPsets for a total of 100 independent subsamples per number of SNP loci being tested. The subsampled SNPsets were further used to calculate pairwise F_ST_ values (using the R package diveRsity) and mean TrioML values (using Coancestry via the R package related)^58^ for each subpopulation.

### Analysis of Inter-Population Gene Flow

To detect the presence of gene flow between subpopulations, we used GENECLASS2^59^ to identify first generation immigrants within each putative subpopulation. We used the L_home likelihood statistic since some source populations may not have been sampled.

### Analysis of Historical Population Demography

To understand the historical demography of the Striped Tit-Babbler population in Singapore, we performed coalescent simulations, compared different models of historical demography, and further estimated parameters in DIYABC^60^. We assigned all samples to a single population and performed simulations to test three population demographic scenarios: (1) the Striped Tit-Babbler population in Singapore has maintained a uniform N_e_, (2) experienced recent decline, and (3) recent expansion (Fig. S14).

We used NeEstimator v2.01^61^ to independently estimate the contemporary N_e_ of Striped Tit-Babblers in Singapore using the linkage disequilibrium method. As N_e_ calculations can simultaneously be biased by the presence of kin and overestimated under aggressive purging of kin^57^, we ran NeEstimator for both the full (n = 46) and kin-filtered (n = 35) SNP matrices without pruning for loci under linkage disequilibrium (5481 loci), to explore the range of possible N_e_ values for the Striped Tit-Babbler population.

### Landscape Connectivity Modelling

To explore changes in forest contiguity over time, we conducted a supervised classification of LandSat 5 TM and LandSat 8 OLI/TIRS imagery (USGS), using the maximum likelihood method in ArcMap 10.0 to produce four land use maps of Singapore for the years 1989, 1997, 2005, and 2013.

We used Circuitscape v4.0.5^62,63^ to model connectivity between extant forest patches in 2013. We defined preliminary resistance parameters of each habitat type along a scale of 1 (no resistance) to 100 (maximum resistance) (Table S6, Supplementary Information) based on the known ecology and habitat requirements of the Striped Tit-Babbler^24,39,64^. We ran Circuitscape v4.0.5 in pairwise mode to generate an exploratory currentmap showing the likely dispersal pathways between habitat fragments.

### Landscape Genomic Analyses

We used GenAlEx^65^ to estimate the extent of spatial autocorrelation between the multilocus genotypes of the individuals sampled, using a distance class size of 1 km for 999 permutations and 1,000 bootstraps. In addition, we conducted distance-based Moran’s eigenvector map (dbMEM) analyses using the R package MEMGENE^66^, which account for potential spatial autocorrelation in the data, to determine the proportion of the spatial genetic signal explained by isolation by distance (IBD) and isolation by resistance (IBR) models.

We refined the resistance model by generating 45 alternative models such that resistance_urban_ >resistance_managedvegetation_, and using Circuitscape v4.0.5 and MEMGENE to select the optimal model for which the proportion of genetic variation explained by the corresponding resistance distance matrix was highest. We used the optimised resistance model to generate the final landscape connectivity map using Circuitscape v4.0.5.

### Forward-in-time Landscape Genomic Simulations

To test for false positive signals of IBR and assess the validity of our population genetic analyses, we used CDPOP^67^ to simulate the effects of landscape structure on the population genetic structure of the Striped Tit-Babbler.

We defined relaxed life history parameters based on the known biology of the Striped Tit-Babbler and closely related species, which should result in a slower accumulation of intrapopulation differentiation, for a spatial dataset of 5761 Striped Tit-Babblers (per the species’ estimated population density, inclusive of the original 46 samples) and simulated the effects of IBD on the population for 99 randomly-generated neutral loci, for 100 generations, sampled every 10 generations, with 100 MCMC replicates per simulation.

We sampled individual genotypes at the locations of the original 46 individuals and calculated the change in pairwise F_ST_ between putative subpopulations using the R package diveRsity for all sampled generations. We also conducted dbMEM analyses using MEMGENE for generations 10 to 50, excluding MCMC replicates for which an entire subpopulation goes extinct, to compare the variance explained by IBD and IBD + IBR models and ascertain the Type I error rate.

### Ethics Statement

We acknowledge the National Parks Board of Singapore for facilitating fieldwork under permit NP/RP13-019-2. All field and lab work was conducted in accordance with regulations outlined by the National University of Singapore’s Office of Safety, Health, and Environment.

## Electronic supplementary material

Supplementary Information

## Data Availability

The Striped Tit-Babbler genome and all short read sequences have been accessioned on both the NCBI Genomes and Short Read Archive with the following BioProject accession number: PRJNA392017. Raw Stacks outputs, CDPOP working files, and custom scripts are available from the corresponding author on request. All other data analysed during this study are included in the Supplementary information files.

## References

[1] Fahrig L (2003). Effects of Habitat Fragmentation on Biodiversity. Annu. Rev. Ecol. Evol. Syst..

[2] Ewers RM, Didham RK (2006). Confounding factors in the detection of species responses to habitat fragmentation. Biol. Rev. Camb. Philos. Soc..

[3] Haddad NM (2015). Habitat fragmentation and its lasting impact on Earth ecosystems. Sci. Adv..

[4] Frankham R (1995). Conservation genetics. Annu. Rev. Genet..

[5] Diamond JM (1972). Biogeographic Kinetics: Estimation of Relaxation Times for Avifaunas of Southwest Pacific Islands. Proc. Natl. Acad. Sci. USA.

[6] Tilman D, May RM, Lehman CL, Nowak MA (1994). Habitat destruction and the extinction debt. Nature.

[7] Brook BW, Bradshaw CJA, Koh LP, Sodhi NS (2006). Momentum Drives the Crash: Mass Extinction in the Tropics. Biotropica.

[8] Kuussaari M (2009). Extinction debt: a challenge for biodiversity conservation. Trends Ecol. Evol..

[9] Brooks TM, Pimm SL, Oyugi JO (1999). Time Lag between Deforestation and Bird Extinction in Tropical Forest Fragments. Conserv. Biol..

[10] Sodhi NS, Liow LH, Bazzaz FA (2004). Avian Extinctions from Tropical and Subtropical Forests. Annu. Rev. Ecol. Evol. Syst..

[11] Ford HA, Walters JR, Cooper CB, Debus SJS, Doerr VAJ (2009). Extinction debt or habitat change? - Ongoing losses of woodland birds in north-eastern New South Wales, Australia. Biol. Conserv..

[12] Gibson L (2013). Near-complete extinction of native small mammal fauna 25 years after forest fragmentation. Science (80-.)..

[13] Lynch, J. F. Responses of breeding bird communities to forest fragmentation. *Nat. Conserv. role remnants Nativ. Veg*. 123–140 (1987).

[14] Adeney JM, Ginsberg JR, Russell GJ, Kinnaird MF (2006). Effects of an ENSO-related fire on birds of a lowland tropical forest in Sumatra. Anim. Conserv..

[15] Edwards DP, Tobias JA, Sheil D, Meijaard E, Laurance WF (2014). Maintaining ecosystem function and services in logged tropical forests. Trends Ecol. Evol..

[16] Huhta, E. & Jokimäki, J. In *Advances of Environmental Sciences* (ed. Daniels, J. A.) 95–111 (Nova Publishers, Inc., 2015).

[17] Harrisson KA (2012). Fine-scale effects of habitat loss and fragmentation despite large-scale gene flow for some regionally declining woodland bird species. Landsc. Ecol..

[18] Peterson, B. K., Weber, J. N., Kay, E. H., Fisher, H. S. & Hoekstra, H. E. Double digest RADseq: An inexpensive method for de novo SNP discovery and genotyping in model and non-model species. *PLoS One* **7** (2012). 10.1371/journal.pone.0037135 PMC3365034 22675423

[19] Manel S, Schwartz MK, Luikart G, Taberlet P (2003). Landscape genetics: Combining landscape ecology and population genetics. Trends Ecol. Evol..

[20] Manel S, Holderegger R (2013). Ten years of landscape genetics. Trends Ecol. Evol..

[21] Moradi HV, Mohamed Z (2010). Responses of Babblers (Timaliidae) to the forest edge – interior gradient in an isolated tropical rainforest in Peninsular Malaysia. J. Trop. For. Sci..

[22] BirdLife International. Macronous Gularis. *IUCN 2013. IUCN Red List of Threatened Species. Version 2013.2* at, http://www.iucnredlist.org/details/22735162/0 (2013).

[23] Cockburn A (2006). Prevalence of different modes of parental care in birds. Proc. R. Soc. B Biol. Sci..

[24] Wells, D. *Birds of the Thai-Malay Peninsula: Passerines: Volume 2*. (Christopher Helm Publishers Ltd, 2007).

[25] O’Dempsey, T. In *Nature Contained: Environmental Histories of* Singapore (ed. Barnard, T. P.) 328 (NUS Press, 2014).

[26] Castelletta, M. Ecology and Conservation of Insular Bird Communities in Fragmented Southeast Asian Forests. (National University of Singapore, 2001).

[27] Frankham R (1995). Efective population size/adult population size ratios in wildlife: a review. Genet. Res..

[28] Rowley, I., Russell, E. & Brooker, M. In *The Natural History of Inbreeding and Outbreeding: Theoretical and Emprical Perspectives* 304–328 (University of Chicago Press, 1993).

[29] Grant PR, Grant BR (1992). Demography and the Genetically Effective sizes of Two Populations of Darwin’s Finches. Ecology.

[30] Waples RS, Gaggiotti O (2006). What is a population? An empirical evaluation of some genetic methods for identifying the number of gene pools and their degree of connectivity. Mol. Ecol..

[31] Benestan, L. *et al*. RAD-genotyping reveals fine-scale genetic structuring and provides powerful population assignment in a widely distributed marine species; the American lobster (Homarus americanus). *Mol. Ecol*. 3299–3315, 10.1111/mec.13245 (2015). 10.1111/mec.13245 25977167

[32] Mastretta-Yanes, A. *et al*. Restriction site-associated DNA sequencing, genotyping error estimation and de novo assembly optimization for population genetic inference. *Mol. Ecol. Resour*. 28–41, 10.1111/1755-0998.12291 (2014). 10.1111/1755-0998.12291 24916682

[33] Tin MMY, Rheindt FE, Cros E, Mikheyev AS (2015). Degenerate adaptor sequences for detecting PCR duplicates in reduced representation sequencing data improve genotype calling accuracy. Mol. Ecol. Resour..

[34] Nazareno, A. G., Bemmels, J. B., Dick, C. W. & Lohmann, L. G. Minimum sample sizes for population genomics: an empirical study from an Amazonian plant species. *Mol. Ecol. Resour*. 10.1111/1755-0998.12654 (2017). 10.1111/1755-0998.12654 28078808

[35] Gelang M (2009). Phylogeny of babblers (Aves, Passeriformes): major lineages, family limits and classification. Zool. Scr..

[36] Khoonwongsa, J. Post Fledging Survival and Juvenile Dispersal in Abbott’s Babbler (Malacocincla abbotti) in Khao Yai National Park. (King Mongkut’s University of Technology Thonburi, 2011).

[37] Meirmans PG (2015). Seven common mistakes in population genetics and how to avoid them. Mol. Ecol..

[38] Kardos M, Taylor HR, Ellegren H, Luikart G, Allendorf FW (2016). Genomics advances the study of inbreeding depression in the wild. Evol. Appl..

[39] Yong DL (2009). Persistence of Babbler (Timaliidae) Communities in Singapore Forests. Nat. Singapore.

[40] Wilcove DS, Giam X, Edwards DP, Fisher B, Koh LP (2013). Navjot’s nightmare revisited: logging, agriculture, and biodiversity in Southeast Asia. Trends Ecol. Evol..

[41] Tay YC (2016). Beyond the CoralTriangle: high genetic diversity and near panmixia in Singapore’s populations of the broadcast spawning sea star Protoreaster nodosus. R. Soc. Open Sci..

[42] Olsen R-A (2015). De novo assembly of Dekkera bruxellensis: a multi technology approach using short and long-read sequencing and optical mapping. Gigascience.

[43] Catchen JM, Amores A, Hohenlohe P, Cresko W, Postlethwait JH (2011). Stacks: building and genotyping Loci de novo from short-read sequences. G3 Genes, Genomes, Genet..

[44] Catchen J, Hohenlohe PA, Bassham S, Amores A, Cresko WA (2013). Stacks: an analysis tool set for population genomics. Mol. Ecol..

[45] Langmead B, Salzberg SL (2012). Fast gapped-read alignment with Bowtie 2. Nat. Methods.

[46] Li H (2011). A statistical framework for SNP calling, mutation discovery, association mapping and population genetical parameter estimation from sequencing data. Bioinformatics.

[47] Foll M, Gaggiotti O (2008). A Genome-Scan Method to Identify Selected Loci Appropriate for Both Dominant and Codominant Markers: A Bayesian Perspective. Genetics.

[48] Chang CC (2015). Second-generation PLINK: rising to the challenge of larger and richer datasets. Gigascience.

[49] Wang J (2011). Coancestry: A program for simulating, estimating and analysing relatedness and inbreeding coefficients. Mol. Ecol. Resour..

[50] Queller DC, Goodnight KF (1989). Estimating Relatedness Using Genetic Markers. Evolution (N. Y)..

[51] Meirmans PG, Van Tienderen PH (2004). GENOTYPE and GENODIVE: Two programs for the analysis of genetic diversity of asexual organisms. Mol. Ecol. Notes.

[52] Wang J (2007). Triadic IBD coefficients and applications to estimating pairwise relatedness. Genet. Res..

[53] Doyle, R. W. Inbreeding and disease in tropical shrimp aquaculture: a reappraisal and caution. *Aquac. Res*. 10.1111/are.12472 (2014).

[54] Dray S, Dufour AB (2007). The ade4 package: implementing the duality diagram for ecologists. J. Stat. Softw..

[55] Bonhomme, V. & Claude, J. Momocs: Outline Analysis Using R. *J. Stat. Softw*. **56** (2014).

[56] Weir BS, Cockerham CC (1984). Estimating F-Statistics for the Analysis of Population Structure. Evolution (N. Y)..

[57] Waples RS, Anderson EC (2017). Purging putative siblings from population genetic data sets: a cautionary view. Mol. Ecol..

[58] Pew J, Muir PH, Wang J, Frasier T (2015). R. related: an R package for analysing pairwise relatedness from codominant molecular markers. Mol. Ecol. Resour..

[59] Piry S (2004). GENECLASS2: A Software for Genetic Assignment and First-Generation Migrant Detection. J. Hered..

[60] Cornuet J-M (2014). DIYABCv2.0: a software to make approximate Bayesian computation inferences about population history using single nucleotide polymorphism, DNA sequence and microsatellite data. Bioinformatics.

[61] Do C (2014). NeEstimator v2: re-implementation of software for the estimation of contemporary effective population size (N e) from genetic data. Mol. Ecol. Resour..

[62] McRae BH, Dickson BG, Keitt TH, Shah VB (2008). Using Circuit Theory to Model Connectivity In Ecology, Evolution, and Conservation. Ecology.

[63] McRae, B. H., Shah, V. B. & Mohapatra, T. K. *Circuitscape4 User Guid*e. at, http://www.circuitscape.org (2013).

[64] Wang, L. K. & Hails, C. J. An Annotated Checklist of the Birds of Singapore. *Raffles Bull. Zool*. Supplement, 1–179 (2007).

[65] Peakall R, Smouse PE (2012). GenALEx 6.5: Genetic analysis in Excel. Population genetic software for teaching and research-an update. Bioinformatics.

[66] Galpern P, Peres-Neto PR, Polfus J, Manseau M (2014). MEMGENE: Spatial pattern detection in genetic distance data. Methods Ecol. Evol..

[67] Landguth EL, Cushman SA (2010). cdpop: A spatially explicit cost distance population genetics program. Mol. Ecol. Resour..

