# Supplementary material for: Novel genome and genome-wide SNPs reveal early fragmentation effects in an edge-tolerant songbird population across an urbanized tropical metropolis
Source: Sci Rep. 2018 Aug 24;8:12804. doi: 10.1038/s41598-018-31074-5 (PMC6109123; doi:10.1038/s41598-018-31074-5)
Supplement: Supplementary file 1 — Supplementary Information [file 41598_2018_31074_MOESM1_ESM.pdf]

Supplementary information

Title: Novel genome and genome-wide SNPs reveal early fragmentation effects in an edge-tolerant songbird population across an urbanized tropical metropolis

David J. X. Tan[1], Balaji Chattopadhyay[1], Kritika M. Garg[1], Emilie Cros[1], Per G. P. Ericson[2], Martin Irestedt[3], Frank E. Rheindt[1]

[1] Department of Biological Sciences, National University of Singapore, 14 Science Drive 4, Singapore 117543
[2] Department of Zoology, Swedish Museum of Natural History, P.O. Box 50007, SE-104 05 Stockholm, Sweden
[3] Department of Bioinformatics and Genetics, Swedish Museum of Natural History, P.O. Box 50007, SE-104 05 Stockholm, Sweden

Extended Materials and Methods

Study Area and DNA Sampling

We conducted the study in Singapore (1.150°-1.483°N and 103.633°-104.100°E; Fig. 1), a 718.3 sq. km island south of Peninsular Malaysia located within the Sundaland biodiversity hotspot in Southeast Asia. Singapore has historically experienced extreme levels of habitat loss and fragmentation owing to agriculture-driven land clearance in the early 19th century followed by rapid urbanisation in the 20th century (Corlett 1992, 1997, O'Dempsey 2014). These changes in land-use have resulted in the loss of over 99% of Singapore's original primary vegetation cover. Today, although approximately 20% of Singapore's land area consists of forest, these forest patches are predominantly broken up into fragments of varying sizes and largely composed of young to maturing secondary regrowth forests embedded within a heavily streetscaped urban landscape matrix, with most forests concentrated in the central and western regions of the island (Fig. 1; Fig. S11).

We conducted targeted mist netting at various forest fragments across Singapore between May 2013 and September 2014, with most mist netting sites clustering around the two major areas of remaining forest fragments in Singapore — the Central Catchment Nature Reserve and the Southern Ridges. We also conducted mist-netting on offshore islands and in isolated forest patches (Fig. 1). We collected blood samples via brachial venipuncture using a fine gauge needle (BD PrecisionGlide, 30 G x 1/2 in). Blood samples were collected using glass capillary tubes (Dummond Microcaps, 50μL) and transferred into either 500μL of Queen's lysis buffer (Seutin et al. 1991) or 50 μL of 100% ethanol topped up to approximately 20x the blood volume. Bleeding was stanched by applying pressure with a cotton swab. All blood samples were stored at 4 °C. In addition, all mist-netted birds were fitted with a uniquely numbered alloy band to enable specimen identification and aid in future recapture studies. All fieldwork was carried out in accordance with national laws and regulations and in close collaboration with the National Parks Board.

Additional DNA samples were obtained from muscle and liver tissues stored at the cryogenic collection of the Lee Kong Chian Natural History Museum as well as from the carcass collection of the NUS Avian Evolution Laboratory. We sampled carcasses by extracting a cube of breast muscle tissue from the specimen with a sterile scalpel. Tissues were either preserved dry or in 100% ethanol and stored at -20 °C. A total of six tissue samples were obtained, five from the cryogenic collection dating to October 2006 and one from the carcass collection from March 2014.

RAD-Seq Library Preparation

For blood samples stored in Queen's lysis buffer, we extracted DNA using the Exgene Clinic SV kit (GeneAll Biotechnology) as per the manufacturer's protocol for blood and body fluid DNA extraction. As for blood samples stored in 100% ethanol, we pelleted the blood in a microcentrifuge at 10,000 RPM before aspirating out the 100% ethanol and leaving the pellet to dry for five minutes. We resuspended the blood pellets in 190μL of phosphate buffered saline (PBS) solution. Subsequent DNA extraction steps followed the manufacturer's protocol for the Exgene Clinic SV kit for blood and body fluid except for an increased proteinase K digestion time to maximize DNA yield. As for muscle and liver tissue samples, we extracted DNA as per the Animal Tissue protocol for the Exgene Clinic SV kit. We eluted the extracted DNA samples into molecular grade water and stored them at -20°C. Fluorometric quantification with the Qubit 2.0 BR DNA Assay (Invitrogen) was used to determine the concentration of DNA for each sample.

We prepared double digest RAD-Seq libraries for each sample based on a modified FASSST protocol as developed by Tay et al. (2016) and Tin et al. (2015). We used combinatorial barcodes derived from Peterson et al. (2012) to tag each individual with a unique adapter sequence, with at least two base-pair differences between each barcode adapter to reduce the likelihood of sample misidentification. The digestion-ligation reaction was conducted as per Tay et al. (2016) using 45 ng of DNA from each sample, and the restriction enzymes EcoRI (NEB)

and MspI (NEB). A total of two reactions were conducted per sample to increase the yield of adapter-ligated restriction fragments. We carried out fragment size selection on the adapter-ligated fragments using Sera-Mag magnetic beads (Thermo Scientific) to select for 300-500 bp long fragments to be used as templates for PCR marker amplification. We amplified the size-selected fragments with two initial PCR extension cycles at 55 °C and 18 subsequent PCR cycles at 68 °C using Q5 High-Fidelity DNA Polymerase (NEB). We conducted triplicate PCR reactions per sample to reduce the likelihood of PCR bias highlighted by Tin et al. (2015) and to maximise the yield of adapter-ligated fragments. We purified the PCR products to select for 300-500 bp long fragments using a second size-selection step with Sera-Mag magnetic beads, and we verified the final fragment size range using a Fragment Analyzer (Advanced Analytical). We quantified the final adapter-ligated fragment concentrations with the Qubit 2.0 BR DNA Assay. In total, we produced 47 successful double digest RAD-Seq libraries, inclusive of one replicate specimen, and pooled them in equimolar volumes. The pooled libraries were sequenced on one lane of an Illumina HiSeq 2000 sequencer at BGI Shenzhen, producing 100 bp paired-end reads.

Whole Genome Sequencing and Assembly

Genomic DNA was extracted from fresh tissue from one Striped Tit-Babbler individual using the KingFisher™ Duo extraction robot (Prime Magnetic Particle Processor) and the KingFisher Cell and Tissue DNA Kit, following the manufacturer´s protocol. Preparation of libraries, sequencing and the assembly of the *de novo* genome were performed by Science for Life Laboratory (SciLifeLab) in Stockholm. Short-insert-sized (180 bp) and mate-pair (5 and 8 kb) DNA libraries were constructed. All libraries were sequenced on the Illumina HiSeq 2500 platform with a 2x126 setup in RapidHighOutput mode. Paired-end sequence data from the genomic DNA libraries were quality-checked, assembled, and evaluated using the NouGAT pipeline (Olsen et al. 2015), which automates the de novo assembly process. To pre-process and ascertain the quality of the sequenced genomic DNA libraries, the NouGAT pipeline uses Trimmomatic (Bolger et al. 2014) and FastQC (Babraham Bioinformatics) to identify and remove low quality and clonally duplicated reads, and generates k-mer counts for the reads using ABySS (Simpson et al. 2009). We subsequently used the NouGAT pipeline to assemble the quality-checked reads using three short oligonucleotide analysis packages, ALLPATHS-LG (Gnerre et al. 2011), ABySS (Simpson et al. 2009), and SOAPdenovo (Li et al. 2010) on default settings. Finally, we evaluated the relative quality of the assemblies using the evaluate pipeline in NouGAT, which computes standard contiguity metrics, contig lengths, and plots feature response curves.

ddRAD-Seq Read Processing and Alignment

We initially analysed the raw sequence reads with FastQC (Babraham Bioinformatics) to determine the average quality scores across all single-end and paired-end reads. We used the *process_radtags* pipeline in Stacks v1.3 (Catchen et al. 2011, 2013) to demultiplex sequence reads and filter out low quality reads. Reads with an average Phred quality score below 20, indicative of a greater than 1% error probability, were discarded from the pool of raw reads. Additionally, *process_radtags* was used to trim all raw sequence reads to 90 bp to further filter out low quality bases at the terminal ends of each read. Single nucleotide errors within the barcode were also automatically corrected by the software. We renamed paired reads using the *bbrename.sh* script from BBMAP v35.10 (http://sourceforge.net/projects/bbmap/) to facilitate read alignment. Although we originally used BWA v0.7.12-r1039 (Li and Durbin 2009) to align the RAD reads to the Striped Tit-Babbler reference genome, we faced significant challenges in removing reads with terminal alignments (where reads are locally mapped with significant portions of both ends soft-masked) as well as unpaired reads, which can contribute to downstream errors in haplotype calling. Hence, we used Bowtie2 v2.2.5 (Langmead and Salzberg 2012) to align the RAD reads to the Striped Tit-Babbler reference genome using the *--very-sensitive* setting for end-to-end read mapping, with the *--qc-filter* and *--no-mixed* options activated to filter out bad reads and ensure that only successfully paired reads were aligned. Successfully aligned reads were processed with SAMtools v1.0 (Li 2011) to filter out reads with a mapping quality score < 25 (corresponding

with a mapping accuracy of 99.7%), exclude improperly paired reads, and convert the SAM files to BAM format.

We used the *ref_map.pl* pipeline in Stacks v1.3 to assemble reference-aligned reads into loci for SNP calling. For the initial assembly step, the minimum stack depth parameter (-m) was set at 11 and the upper error bound for SNP calling set at 0.05 based on optimised exploratory replicate analyses adapted from Mastretta-Yanes et al. (2014) (Figs. S1 – S6). As the original scripts were designed for multiple replicate pairs, the scripts had to be modified to account for the fact that only one replicate pair was used in this study (specimens CSW8313 and PRS3255). Note also that the scripts were written based on Stacks v1.02/1.03 and will not work for more recent versions of Stacks.

We set the -r parameter for the *populations* module to 1 to ensure that only loci with no missing data were reported in the output SNP matrix. To exclude paralogous loci from the final SNP matrix, we manually compiled loci with read counts greater than three standard deviations of the mean read depth into a blacklist, which was passed to the *populations* module for exclusion from the SNP matrix. We further applied a minimum locus depth filter of 20 in *populations* to ensure that output loci had sufficient coverage, and used default parameters in Bayescan and a false discovery rate of 0.05 (Foll and Gaggiotti 2008) to detect loci under selection. To reduce the likelihood of linked loci, the --write-single-snp command was passed to *populations,* and PLINK v1.9 (Chang et al. 2015) was used to identify loci in linkage disequilibrium for subsequent filtering with a window size of 1000, step size of 5, and a VIF threshold of 2. Excluding the one replicate sample, we generated a SNP matrix for 46 Striped Tit-Babbler individuals.

To account for analyses such as Bayesian clustering and principal component analysis (PCA) where kinship bias may exert a significant effect, we used the software Coancestry (Wang 2011) to determine the relatedness coefficients between individuals as well as the inbreeding coefficients for all 46 individuals. We subsequently filtered out one individual from each pair with an inferred relatedness greater than half-sibs (Queller and Goodnight (1989) relatedness coefficient > 0.25) using GenoDive (Meirmans and Van Tienderen 2004), taking care to remove the most interrelated individuals to ensure that the maximum possible number of individuals were retained in the reduced SNP matrix. We used the TrioML method (Wang 2007) in Coancestry to estimate the inbreeding coefficients for each individual as it has been shown to perform better than other estimators, especially for populations with high inbreeding and closely related individuals (Wang 2007, Doyle 2014).

Although sex-biased dispersal may affect the population genetic structure of the Striped Tit-Babbler, the sexually monomorphic nature of the species (Wells 2007) makes it difficult to determine if the species exhibits any form of sex-biased dispersal. However, a review of existing literature indicates the absence of sex-biased dispersal in relatively closely related species such as the Abbott's Wren-Babbler (*Turdinus abbotti*) (Khoonwongsa 2011), which belongs in the sister family Pellorneidae (Gelang et al. 2009), and in Steere's Liocichla (*Liocichla steerii*) (Peng 2006), which belongs in the sister family Leiothricidae (Gelang et al. 2009). As such, we conducted all downstream analyses assuming no sex-biased dispersal in the Striped Tit-Babbler.

<u>Analysis of Population Genetic Structure</u>

We performed a PCA with the kin-filtered SNP matrix using the dudi.pca function in the ade4 package (Dray and Dufour 2007) in R v3.2.2 (R Core Development Team, 2015) to explore the differentiation between individuals within a multivariate framework. The results of the PCA were plotted using the dudi.plot function in the R package Momocs (Bonhomme and Claude 2014).

To calculate genetic diversity statistics, we grouped the 46 samples into three putative subpopulation clusters based on the PCA results (Fig. 2). These clusters include one isolated forest patch (Admiralty Park: n=3), and two large but fragmented networks of forest patches (Southern: n=16, and Central Catchment Nature Reserve: n=27) (Table 2). We conducted an analysis of molecular variance (AMOVA) in GenoDive with 99,999 permutations based on the subpopulation clusters defined to analyse the partitioning of variation within individuals, between individuals, and between subpopulations. We calculated population genetic statistics for both the full (n=46) and kin-filtered (n=35) datasets using the *populations* module in Stacks with and

without the population map flag activated to derive overall and within-subpopulation genetic diversity statistics. We also calculated pairwise Weir and Cockerham's F_{ST} (Weir and Cockerham 1984) using the diffCalc function in the R package diveRsity (Keenan et al. 2013), with confidence intervals calculated using 9,999 bootstraps across loci, despite the small within-subpopulation sample sizes as F_{ST} has been shown to yield accurate estimates for large numbers of loci (Willing et al. 2012). As the removal of kin may on the one hand improve the performance of F_{ST} estimations and on the other hand reduce the precision in F_{ST} (Waples and Anderson 2017), we calculated pairwise F_{ST} values using both the full (n=46) SNP matrix and the kin-filtered (n=35) SNP matrix.

To test the robustness of our population genetic inferences and to assess the informativeness of multilocus SNPs, we used custom bash scripts to randomly subset the original SNP matrix for varying numbers of loci ranging from 2 to 3,500 loci, and recalculated population genetic statistics based on these reduced-loci SNP matrices. We generated 100 independent random subsamples for each number of SNP loci being tested, and each locus-set was passed through the *populations* pipeline in Stacks as a whitelist for a total of 1400 whitelists. The Stacks outputs were further used to calculate pairwise F_{ST} values (using the R package diveRsity) and mean TrioML values (using Coancestry via the R package related (Pew et al. 2015)) for each subpopulation.

<u>Analysis of Inter-Population Gene Flow</u>

To detect the presence of gene flow between subpopulations, we used GENECLASS2 (Piry et al. 2004) to identify first generation immigrants within each putative subpopulation. We used L_home as the likelihood statistic owing to the fact that some source populations may not have been sampled. Assignment tests were performed using the Bayesian method described by Baudouin and Lebrun (2001), and exclusion probabilities were calculated using a Monte-Carlo resampling method following the algorithm described by Paetkau et al. (2004) for 100,000 simulated individuals and an alpha threshold of 0.01.

<u>Analysis of Historical Population Demography</u>

In order to understand the historical demography of the Striped Tit-Babbler population in Singapore, we performed coalescent simulations, compared different models of historical demography, and further estimated parameters in DIYABC (Cornuet et al. 2014). Based on initial results of extent of subdivision within the dataset based on analyses of population genetic structure (see above), we considered all samples as belonging to a single population and performed simulations to test three population demographic scenarios: (1) that the Striped Tit-Babbler population in Singapore has maintained a uniform effective population size (N_e), (2) that the population has experienced a recent decline, and (3) that the population has undergone recent expansion (Fig. 3). We used log-uniform prior distributions for all our parameters and performed a total of 3,000,000 simulations (1 million simulations per scenario). Prior parameter distributions are provided in Table S3 (Supporting Information). We set prior conditions such that present population size should be greater than the historical population size for the contraction scenario and vice versa for the expansion scenario. We chose all four summary statistics estimated in DIYABC for a single population scenario: proportion of monomorphic loci, mean genetic diversity across polymorphic loci (Nei 1987), variance of genetic diversity across polymorphic loci and mean genetic diversity across all loci.

We checked our models by performing a PCA and visually verified if the observed empirical dataset falls within the prior distribution (pre-evaluation of scenarios) using default parameters. We chose the best model by performing a model comparison using direct estimates of model posterior probabilities as well as estimates of posterior probabilities obtained from the logistic regression approach (Fagundes et al. 2007, Beaumont 2008). Further, we obtained posterior predictive error rates and estimated parameter values from the best model. We performed all analyses within DIYABC.

We additionally used NeEstimator v2.01 (Do et al. 2014) to independently estimate the contemporary N_e of Striped Tit-Babblers in Singapore using the linkage disequilibrium method,

with a minimum allele frequency cutoff of 0.02 and a random mating model. As N_e calculations can simultaneously be severely biased by the presence of closely-related individuals, and also overestimated under aggressive purging of kin (Waples and Anderson 2017), we used both the full (n=46) and kin-filtered (n=35) SNP matrices in Stacks and GenoDive without pruning for loci under linkage disequilibrium (containing 5481 loci), to explore the range of possible N_e values for the Striped Tit-Babbler population.

GIS Analysis and Landscape Connectivity Modelling

To explore the changes in forest contiguity over time, we downloaded relatively cloud-free remote sensing images captured by the LandSat 5 TM and LandSat 8 OLI/TIRS sensors from the EarthExplorer LandSat Archive (USGS) to construct land use maps of Singapore. We chose remote sensing images from between the years of 1989 and 2013 as they were the furthest apart available at the time of this study. We subsequently conducted a supervised classification using the maximum likelihood method in ArcMap 10.0 to produce four land use cover maps of Singapore for the years 1989, 1997, 2005, and 2013. We defined training areas based on comparisons with the 2005 land use cover map of Singapore (Yee et al. 2011) and Google Earth data, as well as from habitat information collected during site surveys. We defined a total of seven land-use types (Table S5, Supporting information) based on the known habitat preferences of the Striped Tit-Babbler. To remove noise and isolated pixels, we filtered the classified output using the majority filter tool in ArcMap 10.0. Two additional similarly classified images from 2014 were composited onto the 2013 map to correct for cloud cover (Table S4, Supporting information).

We used Circuitscape v4.0.5 (McRae et al. 2008, 2013), which applies circuit theory to infer multiple dispersal pathways between habitat nodes, to model the connectivity between extant forest patches in 2013. To generate the input habitat focal node files and resistance grid files, we used the Gnarly Landscape Utilities ArcGIS toolbox (McRae et al. 2014) in ArcMap v10.0 to create a raster grid of core habitat localities based on the present extent of forest and woodland cover, as well as a preliminary resistance surface grid. We defined the initial resistance parameters of each habitat type along a scale of 1 (no resistance) to 100 (maximum resistance) (Table S5, Supporting Information) based on the known ecology and habitat requirements of the Striped Tit-Babbler (Wang and Hails 2007, Wells 2007, Yong 2009). We ran Circuitscape v4.0.5 in pairwise mode to generate an exploratory currentmap showing the likely dispersal pathways between habitat fragments.

Landscape Genomic Analyses

To explore the spatial genetic structure of the Striped Tit-Babbler, used GenAlEx (Peakall and Smouse 2012) to estimate the extent of spatial autocorrelation between the multilocus genotypes of the individuals sampled. We used a distance class size of 1 km for a total of 15 distance classes to maximise the number of samples per distance bin, and ran the spatial autocorrelation analysis using 999 permutations with 1,000 bootstraps. In addition, we conducted distance-based Moran's eigenvector map (dbMEM) analyses, which account for potential spatial autocorrelation in the data and have been shown to be more powerful and informative than Mantel tests at revealing weak and cryptic spatial genetic structure (Galpern et al. 2014, Legendre et al. 2015) to determine the proportion of the spatial genetic signal explained by isolation by distance (IBD) and isolation by resistance (IBR) models. We calculated Bray-Curtis (1957) (Bray and Curtis 1957) genetic distances (Legendre and Legendre 1998) for each pairwise combination of Striped Tit-Babbler individuals owing to the ease of computation as well as its correlation with other genetic distance metrics such as Rousset's a_r (Cushman et al. 2006) using the R package ecodist (Goslee and Urban 2007). We subsequently used the mgLandscape() function in the R package MEMGENE (Galpern et al. 2014) to compare the explanatory power of the Euclidean distance matrix (corresponding with IBD) and the preliminary resistance model (corresponding with IBD+IBR). This function computes a least-cost distance matrix from the resistance model and the coordinates of the sampled individuals, and performs a redundancy analysis using the spatial MEM eigenvectors extracted from the Euclidean and least-cost distance matrices against

the Bray-Curtis genetic distance matrix for 1,000,000 forward and final permutations, followed by variance partitioning to determine the proportion of variation explained by spatial and non-spatial factors for each model.

Based on the dbMEM results, we further refined the resistance model by varying the resistance values for the urban and managed vegetation habitat strata between 10 and 100 in increments of 10, keeping all other parameters constant, and generating a resistance surface grid for every pairwise combination of values where $resistance_{urban}$ > $resistance_{managedvegetation}$ (since parks and gardens are known to be less resistant to Striped Tit-Babbler movements than urban areas). We generated a total of 45 alternative landscape resistance models. To choose the optimum resistance model, we ran each alternative resistance grid in Circuitscape, using the capture locality coordinates of the sampled individuals as focal nodes, to obtain 45 pairwise resistance distance matrices for all 46 Striped Tit-Babbler samples. Using the resistance distance matrices as input instead of least-cost distances, we used MEMGENE to compare the amount of spatial genetic variation explained by each alternative resistance model for 1,000 forward and final permutations, and selected the model for which the proportion of genetic variation explained by spatial predictors was highest. We subsequently validated the final optimised resistance model using the mgLandscape() function in MEMGENE, with the default least-cost distance as input, to compare its performance against the Euclidean distance null model and the initial preliminary resistance model, with 1,000,000 forward and final permutations. We used the optimised and validated resistance model to generate the final landscape connectivity map using Circuitscape v4.0.5.

Forward-in-time Landscape Genomic Simulations

To test for false positive signals of IBR and to assess the validity of our population genetic analyses, we used CDPOP (Landguth and Cushman 2010) to simulate the effects of landscape structure and differing landscape genetic models on the population genetic structure of the Striped Tit-Babbler in Singapore. To simulate the Striped Tit-Babbler population in Singapore, we used QGIS to extract a clipped polygon vector shapefile of forest and woodland patches along the central north-south axis of Singapore from the 2013 classified land use map generated from LandSat data. We subsequently used the Random Points tool in QGIS to generate 5715 simulated Striped Tit-Babblers (based on the estimated population density of the species for Singapore) randomly distributed within the boundaries of the forest/woodland polygons, giving a spatial dataset of 5761 individual Striped Tit-Babbler locations. To simulate the effects of IBD, we calculated pairwise Euclidean distances between each of the 5761 individuals using the distance() function in the ecodist package (Goslee and Urban 2007).

Based on the known biology of the Striped Tit-Babbler and reasonably closely related species, we defined relatively relaxed life history parameters coding for high maximum longevity (16 years, based on the longevity of the Jungle Babbler (*Turdoides striata*) (Brown 1928)), moderately low fledgling mortality (30%) and low adult mortality (14% per age class) (Fogden 1972), delayed adult maturation due to cooperative breeding habits (Cockburn 2006), and a mean of three offspring per generation (Wells 2007). Based on the results of the spatial autocorrelation analysis we set the maximum threshold of offspring dispersal at 1 km, with an inverse square dispersal distance probability. As no information exists to suggest sex-biased dispersal for the Striped Tit-Babbler or in relatively closely related species such as the Abbott's Wren Babbler (*Turdinus abbotti*) (Khoonwongsa 2011) (family Pellorneidae) or Steere's Liocichla (*Liocichla steerii*) (Peng 2006) (family Leiothricidae), we assumed no sex-biased dispersal in this simulation. The mean neutral SNP mutation rate was estimated based on the galliform mutation rate of 1.8×10^{-9} sites per year (Axelsson et al. 2004).

We allowed CDPOP to generate random genotypes for each individual at the start of the simulation to maximise genetic diversity, and configured the simulation for 99 neutral loci based on the method proposed by Kimura and Crow (1964) for estimating minimum effective alleles (Kimura and Crow 1964, Schmidt et al. 2017). We ran the IBD simulations for 100 generations, sampled every 10 generations, with 100 MCMC replicates per simulation.

To test for false positive signals of IBR, we sampled individual genotypes at the locations of the original 46 individuals from the IBD simulations for generations 10 to 50, excluding

MCMC replicates for which an entire subpopulation goes extinct. We conducted a dbMEM analysis using MEMGENE for each generation to compare the amount of variance explained by the IBD-only Euclidean distance matrix and the IBD+IBR least cost distance matrix, with the expectation that the IBD-only distance matrix would explain a greater proportion of genetic variation for an IBD-only simulation. We summarised the results across the MCMC replicates and calculated the proportion of simulations with false positive results.

In addition, to assess the validity of our empirically derived F_{ST} estimates, we calculated the change in pairwise F_{ST} values between putative subpopulations over time for the IBD simulations. We sampled individual genotypes at the localities of the original 46 individuals for all sampled generations, discarding MCMC replicates if at least one subpopulation goes extinct, and used diveRsity to calculate pairwise F_{ST} values.

***de novo* assembly parameter optimisation**

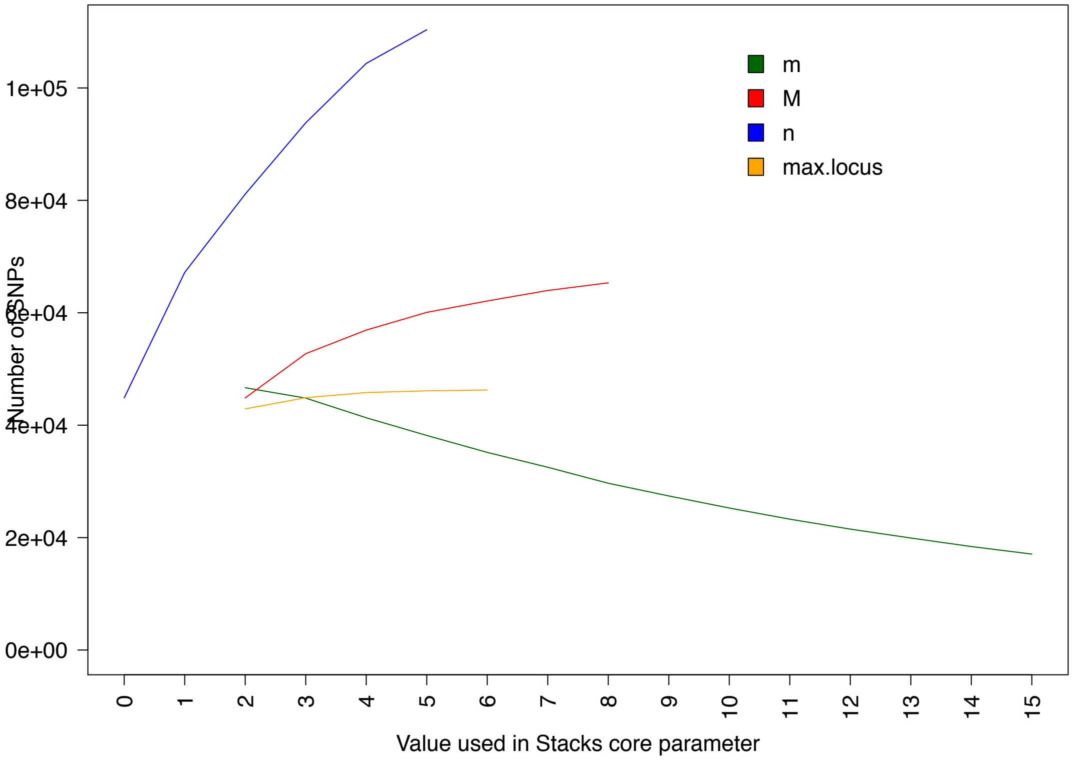

Figure S1: Graph showing the change in the number of SNPs recovered for different Stacks assembly parameter values. The results show that relaxing the mismatch parameters for stacks (M) and for catalog loci (n) resulted in an increase in the number of SNPs recovered. Likewise, increasing the number of stacks at a single *de novo* locus (max.locus) results in an increase in the SNP yield. Conversely, increasing the minimum stack depth parameter (m) results in a decrease in the number of SNPs recovered from the RAD reads.

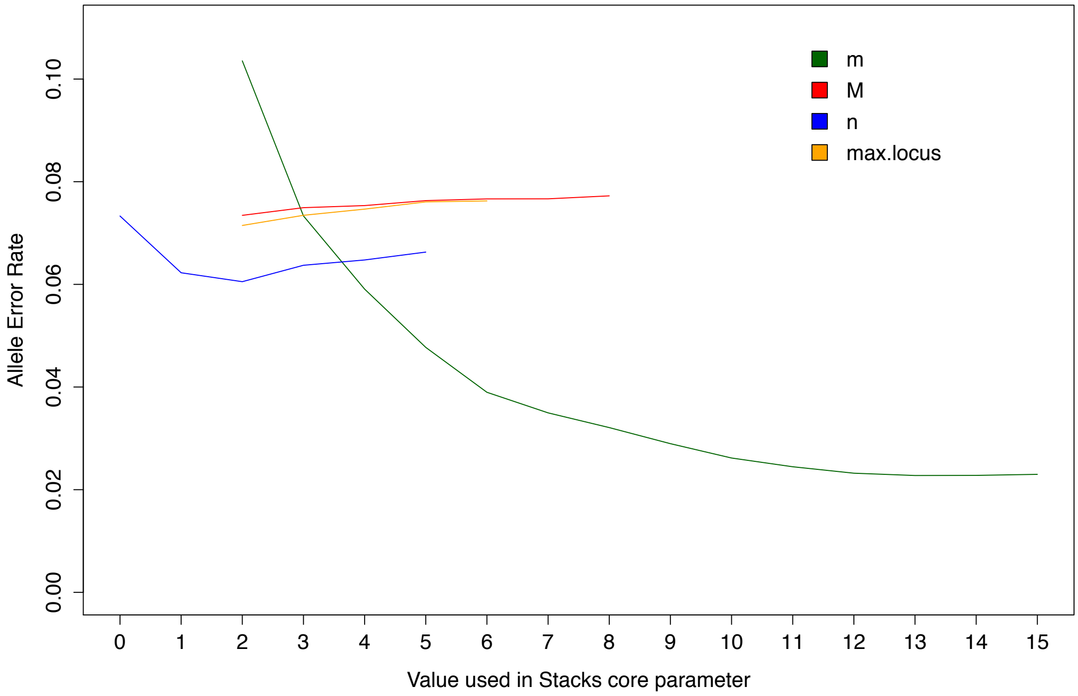

Figure S2: Graph showing the change in allele error rate for different Stacks assembly parameter values, corresponding with the number of allele mismatches between replicate pairs divided by the number of loci being compared. The results show that the allele error rate decreases as the minimum stack depth parameter (m) increases, while the allele error rate increased as both the distance between stacks (M) and maximum number of stacks at a single locus (max.locus) parameters increased. As for the distance between catalog loci parameter (n), the results show an optimal range at n = 1 and n = 2 where allele error rates are minimised.

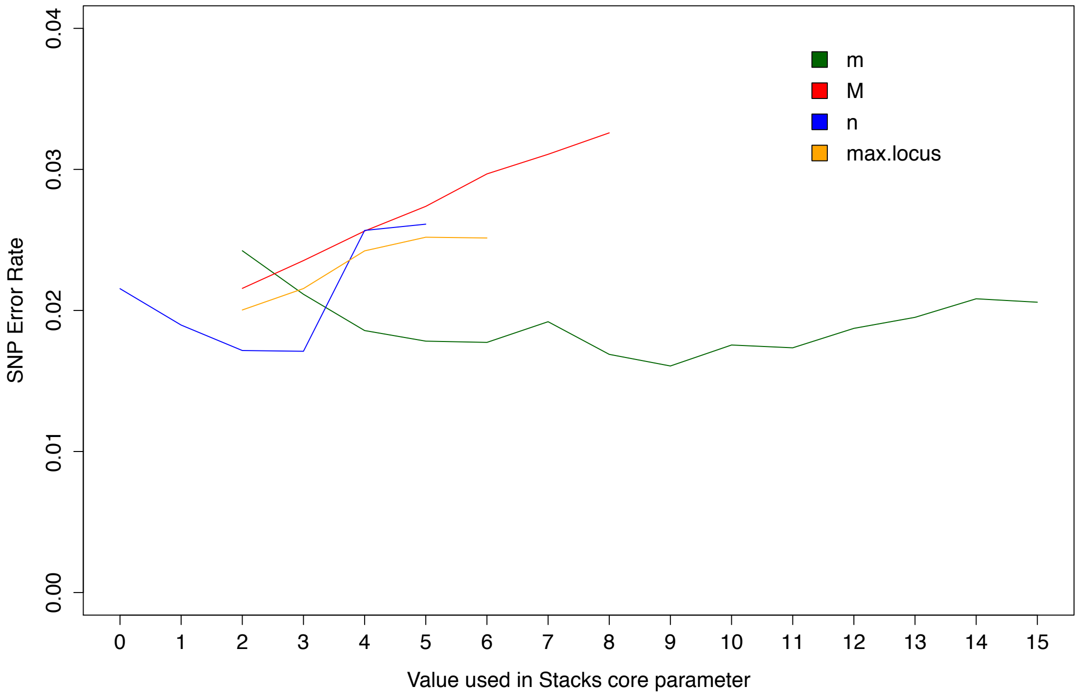

Figure S3: Graph showing the change in SNP error rates, which represents the proportion of SNP mismatches between a replicate pair, for different Stacks assembly parameter values. The results show that increasing the distance between stacks (M) and maximum number of stacks per locus (max.locus) parameters gives rise to a corresponding increase in the SNP error rate. For both the distance between catalog loci (n) and minimum stack depth (m) parameters, the results show a range of optimal values (n = 2 or 3; m = 8 – 11) for which SNP error rates are minimised.

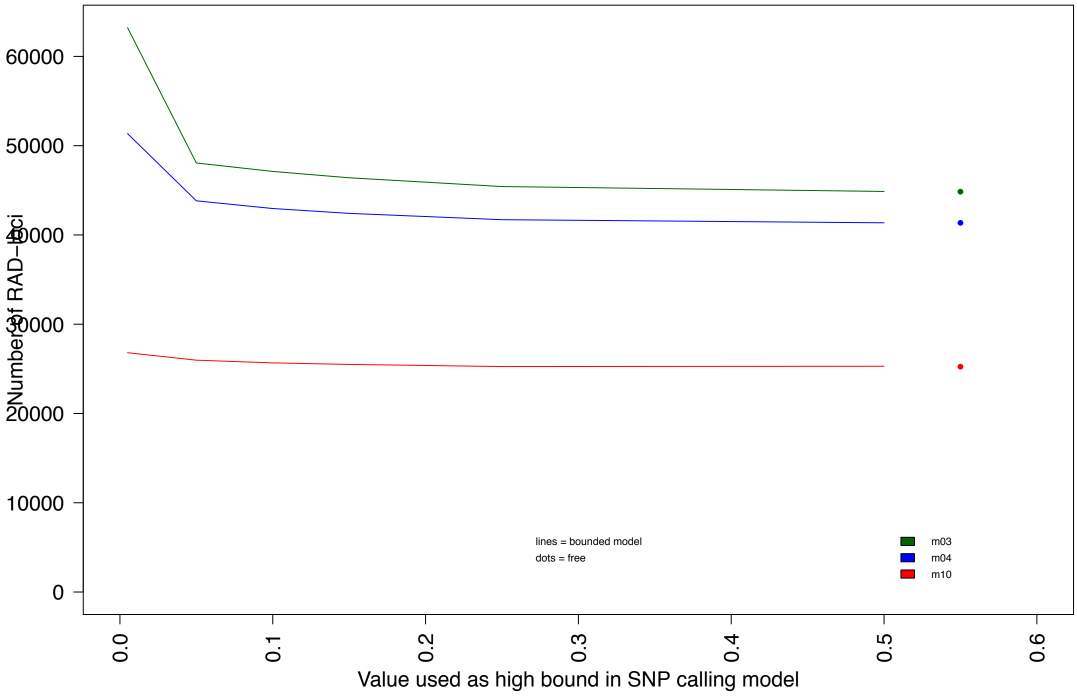

Figure S4: Graph showing the change in the number of loci recovered when varying the upper error bound of the SNP calling model for three values of the minimum stack depth parameter (m). The results show that increasing the upper bound of the SNP calling model results in a reduction of the total number of loci recovered from the RAD reads.

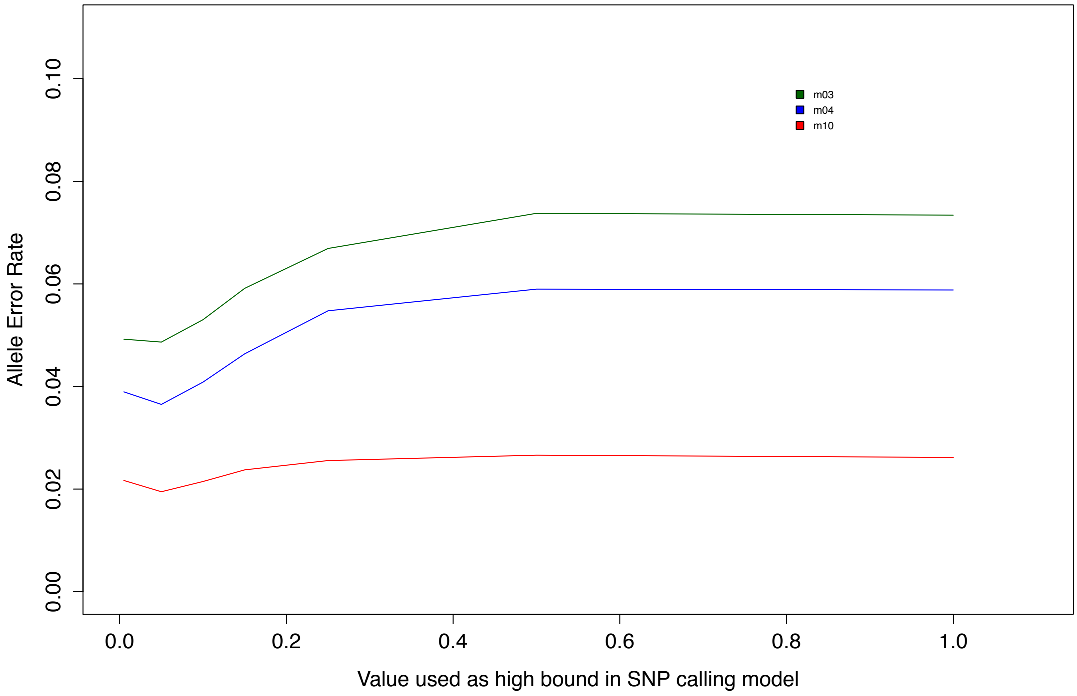

Figure S5: Graph showing the change in the allele error rate, corresponding with the number of allele mismatches between replicate pairs divided by the total number of loci compared, for different upper error bound values for the SNP calling model. This was repeated for three values of the minimum stack depth parameter (m) to infer trends in the allele error rate. The results show that the allele error rate is optimised at an upper error bound of 0.05.

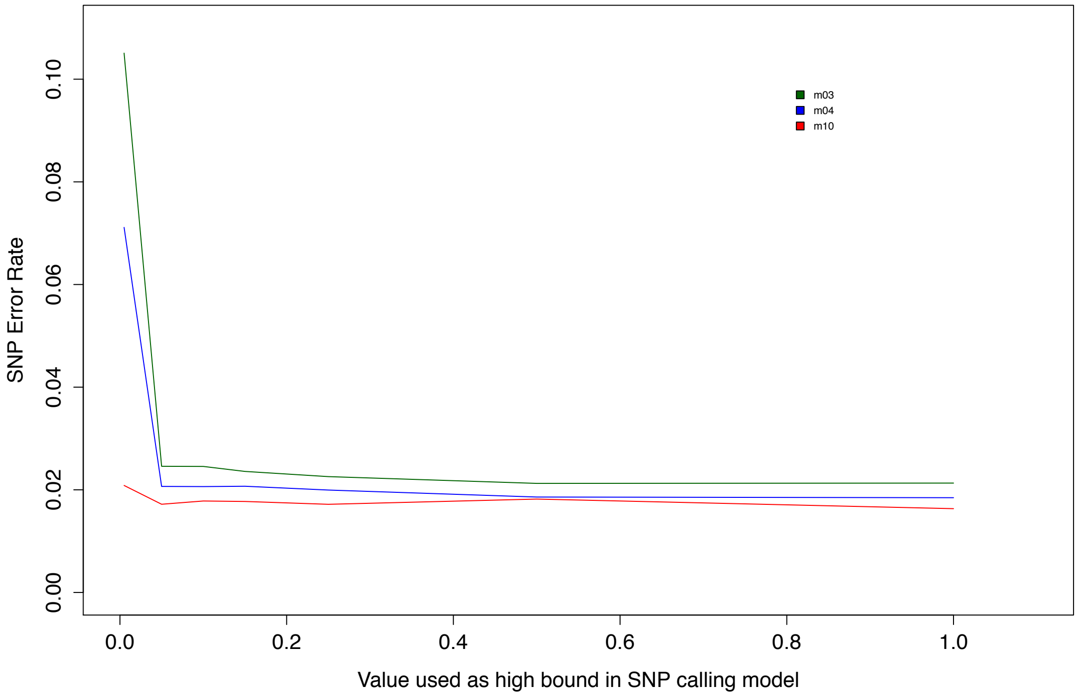

Figure S6: Graph showing the change in the SNP error rate, corresponding with the proportion of SNP mismatches between a replicate pair, for different upper error bound values for the SNP

calling model. This was repeated for different values of the minimum stack depth parameter (m) to determine common response trends. The results show that the SNP error rate is relatively stable for any upper error bound value higher than 0.05, and that values less than 0.05 are likely to result in unreasonably high SNP error rates.

Genome assembly details

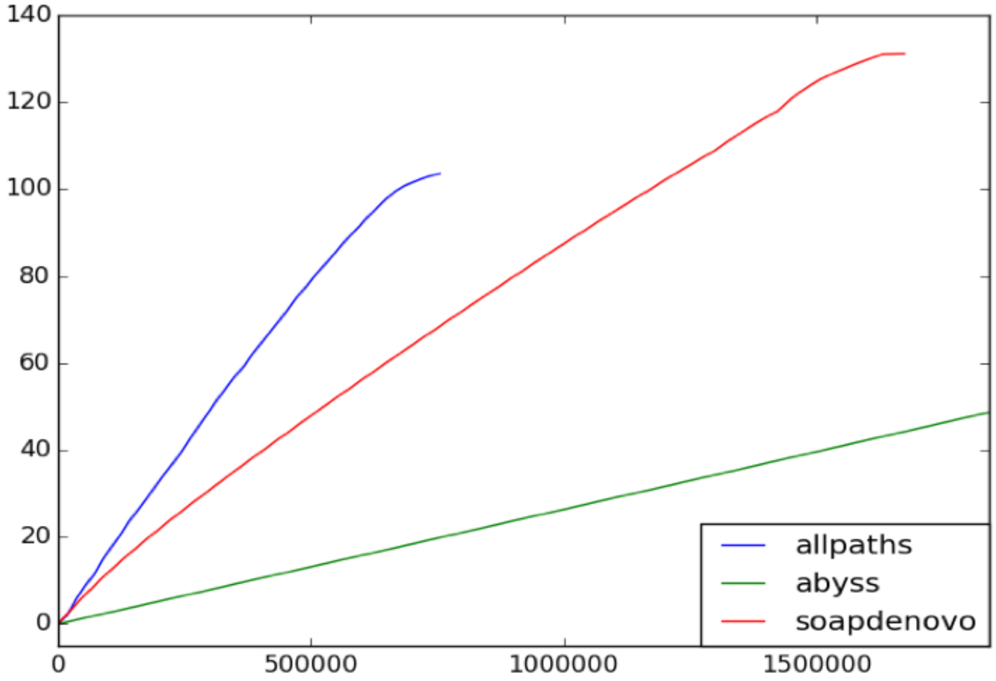

Figure S7: Feature-Response curves for the genome assemblies produced by ALLPATHS-LG, ABySS, and SOAPdenovo, with the total number of features plotted on the x-axis and the coverage of each feature plotted on the y-axis. As can be observed in the figure, the ALLPATHS-LG curve exhibits the highest coverage for the least number of features, indicating that a higher portion of the genome is assembled with fewer errors relative to the other assemblers, showing that the ALLPATHS-LG assembly is the optimal genome assembly.

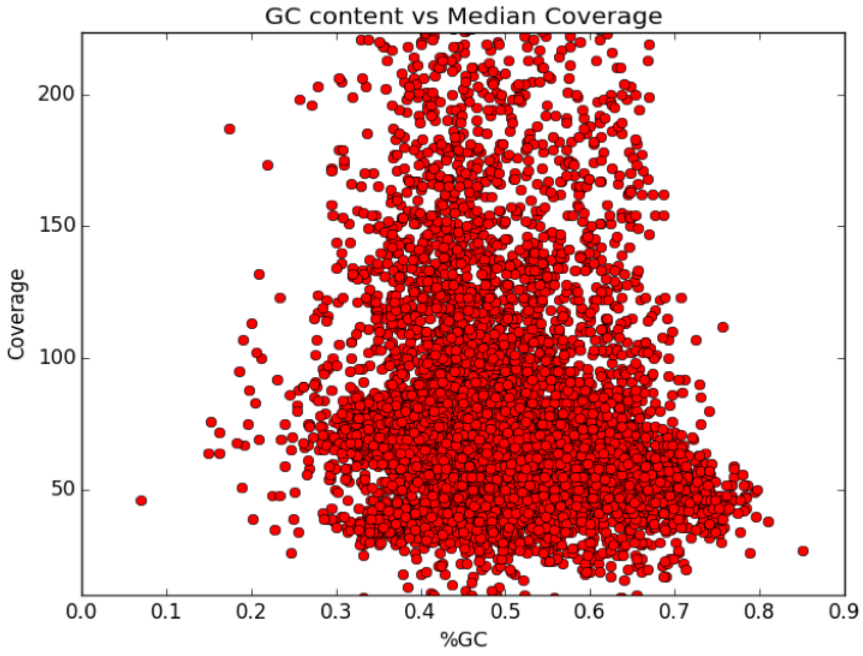

Figure S8: %GC content for each scaffold against median coverage for the ALLPATHS-LG assembly.

Specimen information and read counts for both raw and mapped reads

Table S1: Summary of the 46 samples used, including the specimen IDs, sampling localities, DNA source, as well as the number of RAD reads before and after processing. Excluded from this table is the replicate sample (specimen ID PRS3255), which is a replicate of sample CSW8313. This table also includes the number of reads mapped to the reference genome by both BWA and Bowtie2, showing that more reads were mapped to the reference genome with BWA compared to Bowtie2. However, it should be noted that the reads mapped by BWA also included soft-masked locally mapped reads as well as unpaired reads, which would have contributed toward downstream errors in haplotype calling.

Sample	Locality	DNA Source	Total Reads	Retained Reads	Mapped Reads - BWA	Mapped Reads-Bowtie2
K1103	Admiralty Park	Blood	7240494	6524291	5509357	4579356
K1104	Admiralty Park	Blood	8513906	7414164	6305152	5212580
K1105	Admiralty Park	Blood	7326138	6674555	5639967	4765918
J2843	Central Catchment	Blood	6472652	5573451	4617858	3868034
J3137	Central Catchment	Blood	6618812	5708010	4774918	4032082
J3295	Central Catchment	Blood	6710982	5699142	4784457	4015220
J3502	Central Catchment	Blood	6680370	5896418	4862404	4123518
K0626	Central Catchment	Blood	7297272	6323179	5356690	4524690
K0628	Central Catchment	Blood	7774964	7123158	6087057	5085034
K0630	Central Catchment	Blood	9016960	8021007	6692807	5580514
K0636	Central Catchment	Blood	6995226	6353741	5414337	4527166
K0640	Central Catchment	Blood	6783212	6225503	5368240	4633976
K0646	Central Catchment	Blood	9383196	8525096	7099347	5968108
K0653	Central Catchment	Blood	7404726	6048030	4977436	4143608
K0655	Central Catchment	Blood	9201374	8261106	6773726	5629898
K0660	Central Catchment	Blood	7003862	6332772	5442726	4559256
K0662	Central Catchment	Blood	7504790	6471148	5017219	4090536
K0670	Central Catchment	Blood	12916628	11433142	9516014	7998838
K0673	Central Catchment	Blood	8091290	7462186	6440245	5577166
K0674	Central Catchment	Blood	7178734	6414664	5476001	4667548
K0676	Central Catchment	Blood	6172182	5355005	4380558	3666228
K0679	Central Catchment	Blood	7602744	6842722	5775150	4957800
K0682	Central Catchment	Blood	16594764	14316443	11766877	9898116

K0683	Central Catchment	Blood	9051728	7989425	6463443	5425004
K0684	Central Catchment	Blood	6831700	6047947	5054040	4279926
K0685	Central Catchment	Blood	7210410	6504765	5490611	4649822
K1107	Central Catchment	Blood	9084988	7712125	6551324	5558486
K1109	Central Catchment	Blood	7621344	6865695	5712975	4813326
K1111	Central Catchment	Blood	4698714	4225764	3531875	3017218
L1548	Central Catchment	Blood	9495550	8662943	7423711	6308784
K1118	Sentosa	Blood	6723206	6041323	4996147	4182358
K1119	Sentosa	Blood	7282860	6337641	5263924	4435934
K1120	Sentosa	Blood	6399636	5443494	4362062	3647136
CR062	Southern Ridges	Carcass	7909572	7109259	5003712	4167432
K1102	Southern Ridges	Blood	7744484	7063073	6002704	5082558
K1106	Southern Ridges	Blood	6295924	5569484	4560274	3914484
K1112	Southern Ridges	Blood	8566456	7726524	6519325	5560442
K1113	Southern Ridges	Blood	7276486	6642974	5577297	4701604
K1114	Southern Ridges	Blood	8423230	7637889	6514617	5482852
K1115	Southern Ridges	Blood	7288480	6330749	5398424	4547728
K1116	Southern Ridges	Blood	7390280	6516729	5569250	4735128
K1117	Southern Ridges	Blood	7121090	6283143	5346736	4510472
CSW8311	Southern Ridges	Tissue	7314008	6475509	5003712	4167432
CSW8312	Southern Ridges	Tissue	5618224	4848889	3809033	3137342
CSW8313	Southern Ridges	Tissue	13015174	11012305	9156787	7666862
KEL417	Southern Ridges	Tissue	7084224	5568890	4459162	3726540

Kinship and Inbreeding Analysis

Table S2: Summary of the COANCESTRY analysis, indicating which specimens were filtered out as a result of high relatedness values (>0.25 Queller & Goodnight relatedness coefficient). 'Yes' indicates that the sample was excluded from the reduced SNP dataset. We also report the TrioML inbreeding coefficients for each individual.

Sample	Kin-filtered	TrioML Inbreeding Coefficient
K1103	No	0.1597
K1104	No	0.0567
K1105	Yes	0.0861
J2843	Yes	0.0533
J3137	No	0.0001
J3295	No	0.0005
J3502	Yes	0.0074
K0626	Yes	0.0114
K0628	No	0.0039
K0630	Yes	0.0003
K0636	No	0.002
K0640	No	0.0005
K0646	No	0.0011
K0653	No	0.0009
K0655	No	0.0031
K0660	No	0.0005
K0662	No	0.0385
K0670	Yes	0.0001
K0673	No	0.0248
K0674	No	0.004
K0676	Yes	0.0003
K0679	No	0
K0682	No	0.0104
K0683	Yes	0.0164
K0684	No	0.0383
K0685	No	0.002
K1107	No	0.0019
K1109	No	0.0223
K1111	No	0.003
L1548	No	0.0006
K1118	No	0.0055
K1119	Yes	0
K1120	No	0.0229
CR062	No	0.0122
K1102	No	0.0815
K1106	No	0.0367
K1112	No	0.0216
K1113	No	0.0136
K1114	No	0.0227
K1115	No	0.0004
K1116	Yes	0.0015
K1117	No	0.0207
CSW8311	No	0.0012
CSW8312	No	0.0059
CSW8313	No	0.011
KEL417	Yes	0.0007

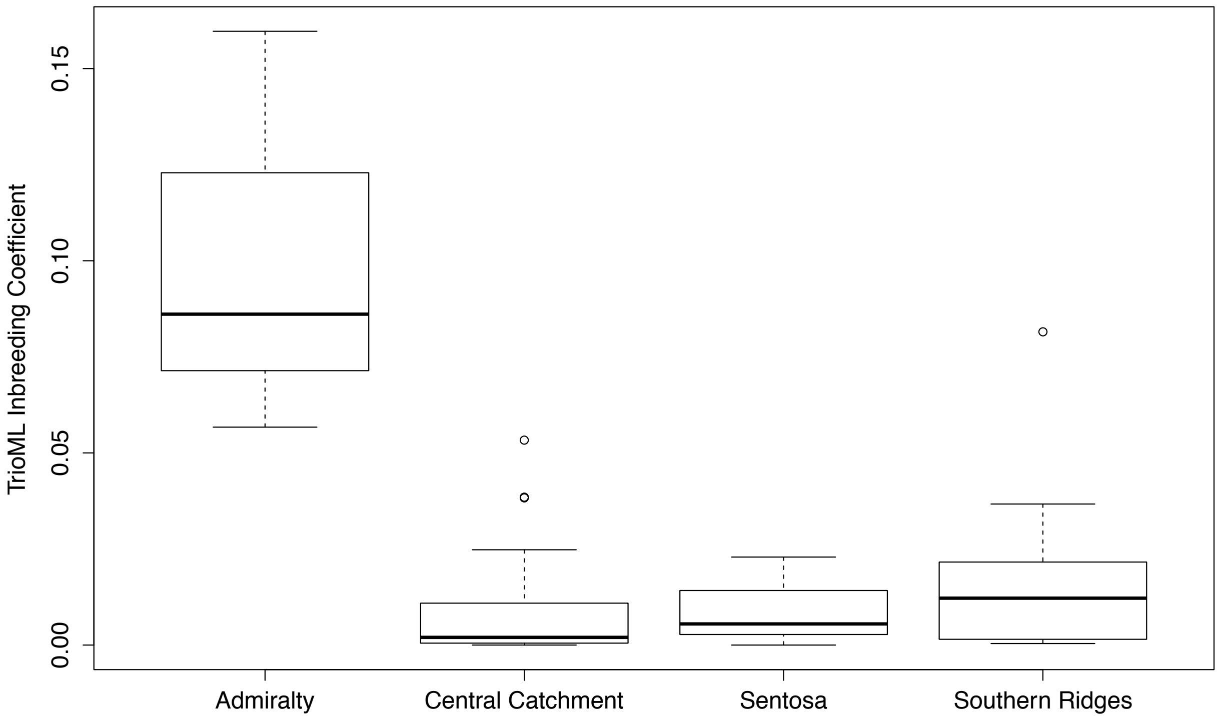

Figure S9: Boxplot showing the spread of TrioML individual inbreeding coefficient values for each population, demonstrating that the Admiralty Park population is significantly more inbred than all the other surveyed populations.

Tests of parameter precision

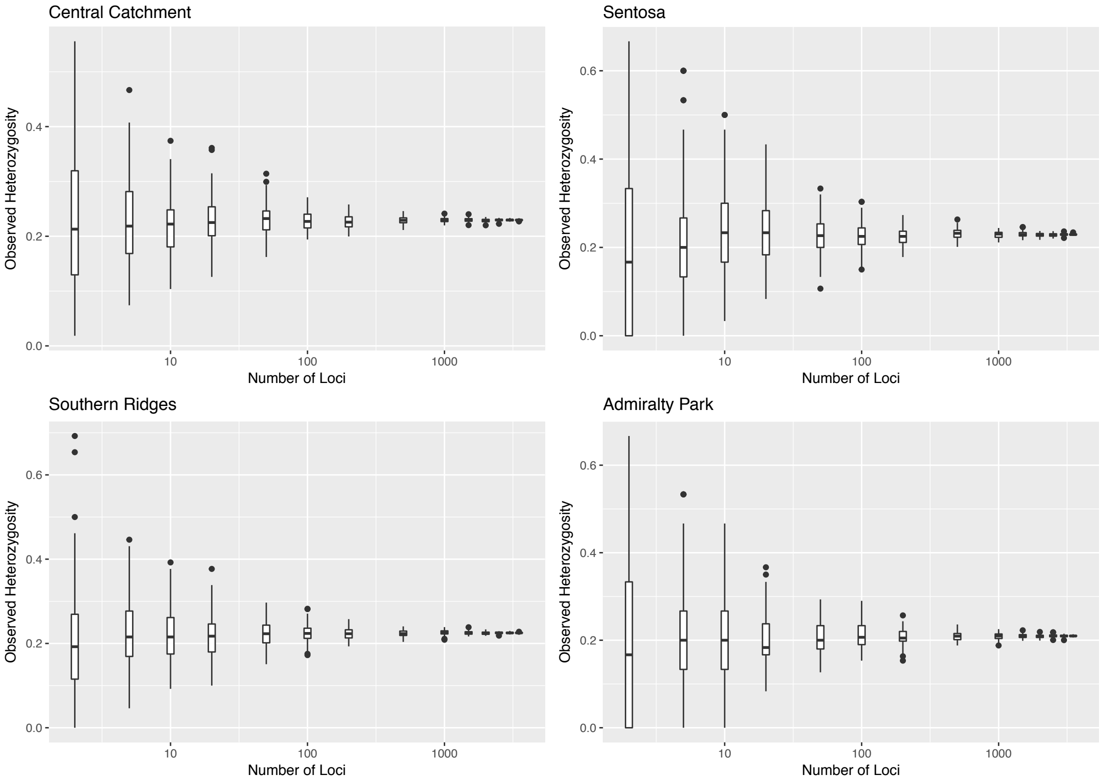

Figure S10: Effect of number of SNP loci on the mean observed heterozygosity estimates obtained for each putative Striped Tit-Babbler subpopulation. The results show that observed heterozygosity values exhibit relatively high levels of precision when more than 500 SNP loci are used.

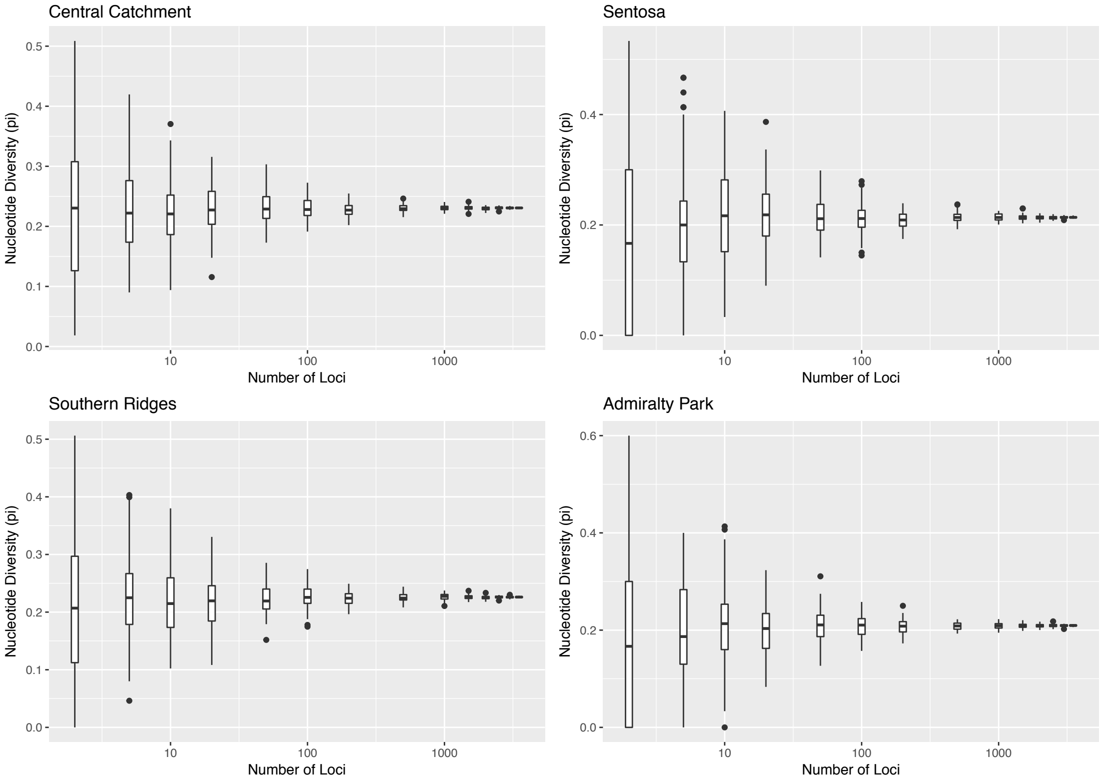

Figure S11: Effect of number of SNP loci on the mean nucleotide diversity estimates obtained for each putative Striped Tit-Babbler subpopulation. The results show that nucleotide diversity values exhibit relatively high levels of precision when more than 500 SNP loci are used.

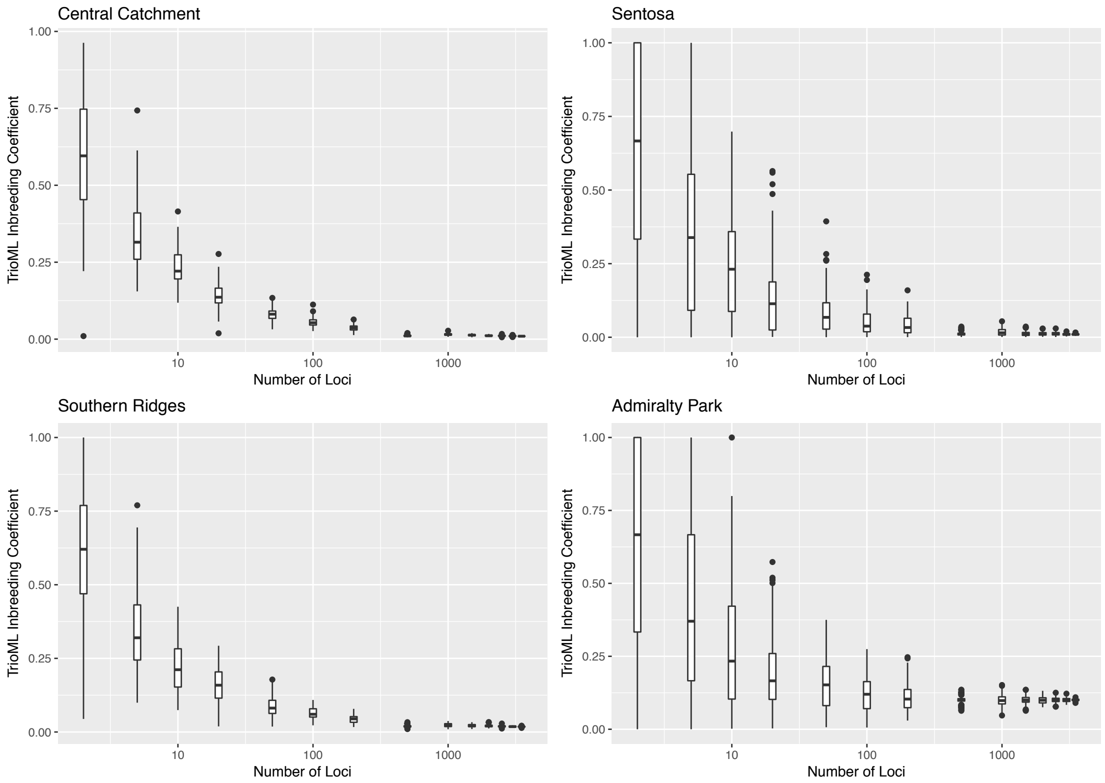

Figure S12: Effect of number of SNP loci on the mean TrioML inbreeding coefficient estimates obtained for each putative Striped Tit-Babbler subpopulation. The results show that TrioML inbreeding coefficients exhibit relatively high levels of precision when more than 500 SNP loci are used.

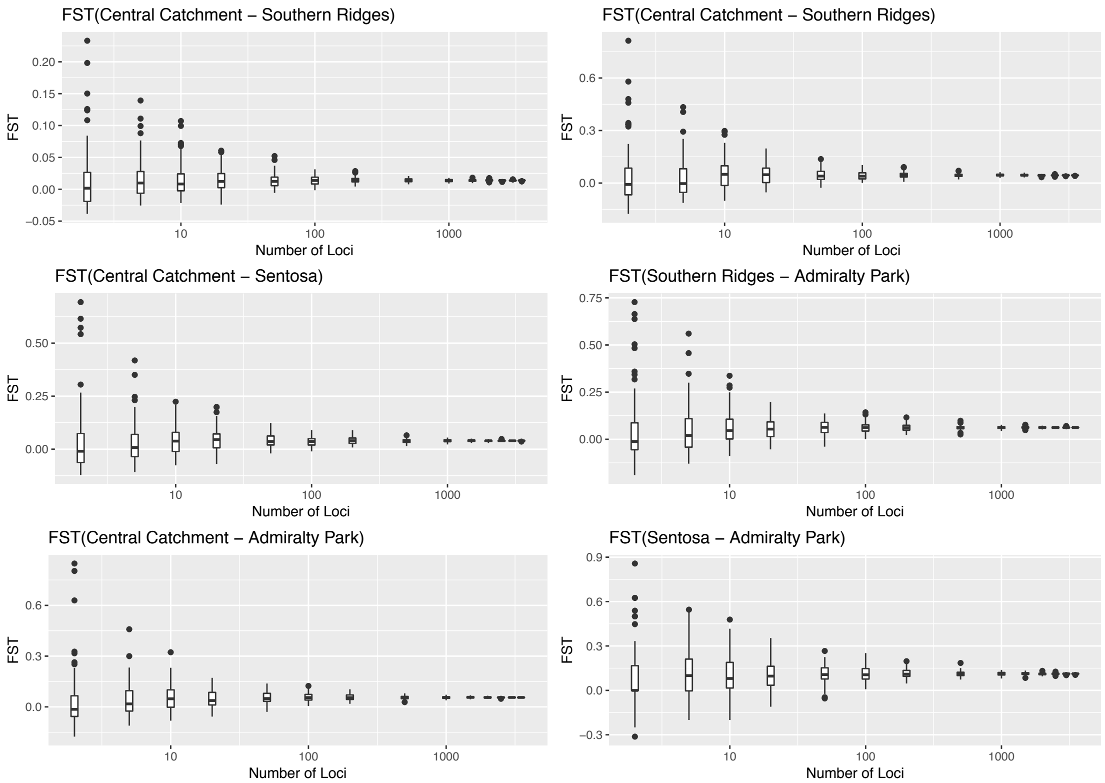

Figure S13: Effect of number of SNP loci on the mean pairwise F_{ST} estimates obtained for each putative Striped Tit-Babbler subpopulation. The results show that pairwise F_{ST} values exhibit relatively high levels of precision when more than 500 SNP loci are used.

Demographic history modeling using DIYABC

Table S3: Prior distribution of parameters present in models compared using DIYABC

Parameter	Lower Bound	Upper Bound
N	10	1000000
N_{dec}	10	1000000
N_{exp}	10	1000000
t	5	100000

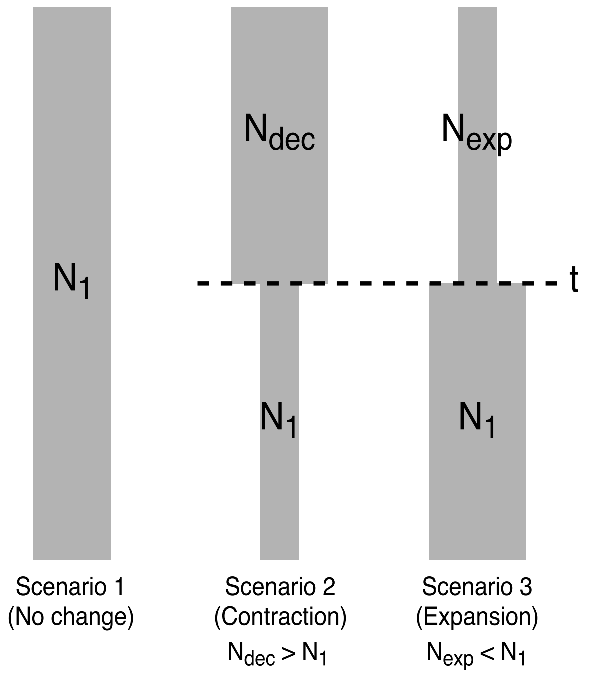

Figure S14: Scenarios of population demographc history tested using DIYABC. Scenario 1 represents a population of uniform size with no change in population size over time. Scenario 2 represents a contraction scenario, with the historical effective population size (N_{dec}) undergoing a contraction to a population of size N at time t. Scenario 3 represents an expansion scenario, with a historical population N_{exp} undergoing an expansion to a population of size N at time t.

DIYABC Outputs

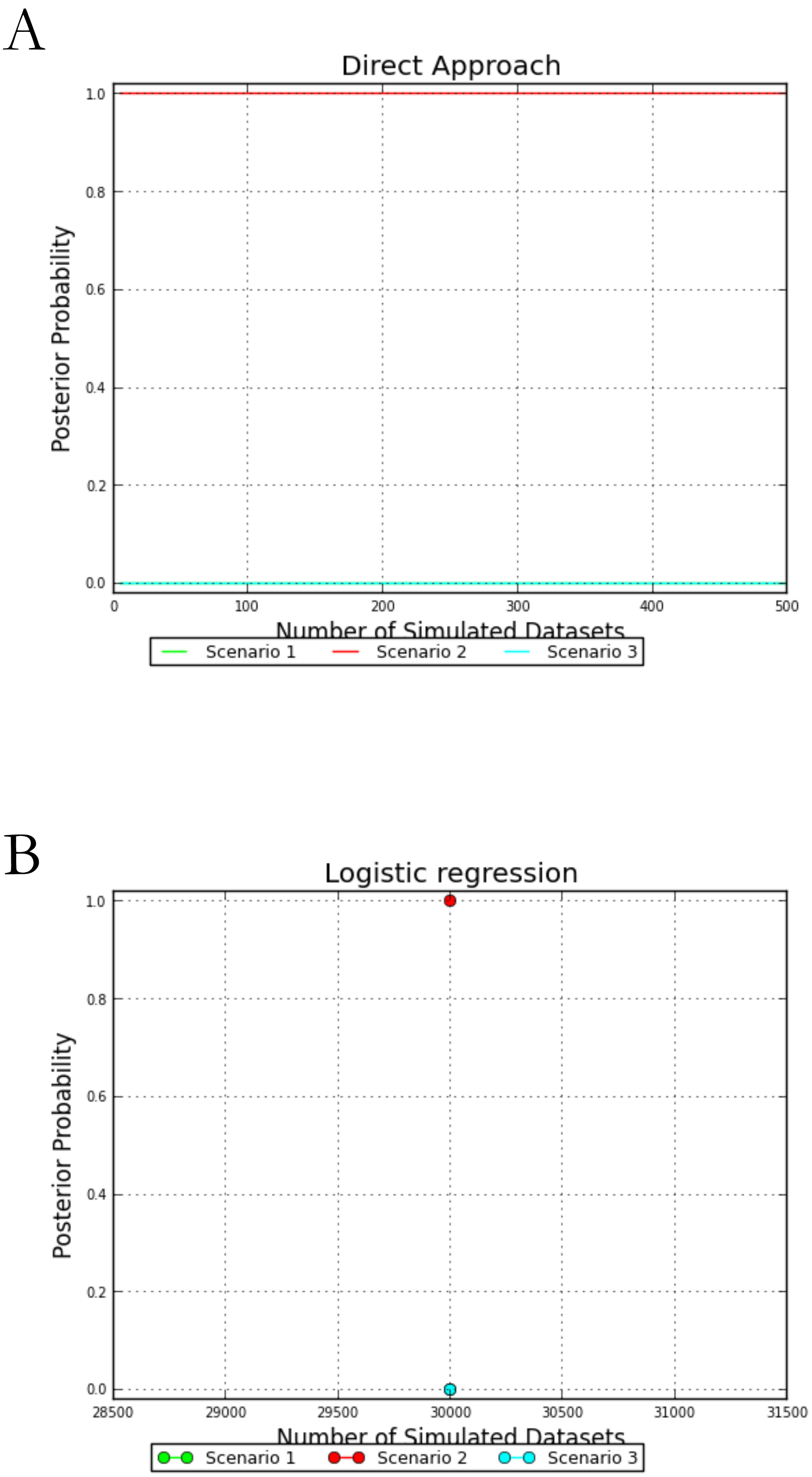

Figure S15: Model comparison of population demographic history scenarios (Fig. 3) using (A) a direct approach, and (B) using a logistic regression. The model comparisons clearly show that Scenario 2 (the contraction scenario) is the scenario with the best support.

Table S4: Confidence estimates of the demographic parameters estimated using DIYABC under Scenario 2 (population contraction), for median and mode point estimates. We report the mean relative bias (MRB), the root of the relative mean square error (RRMSE), and the relative median of the absolute error (RMAE) for each parameter estimate. The overall low error associated with each parameter estimate suggests that Scenario 2 performs well for estimating posterior distributions of parameters, and while a greater degree of error is associated with the median point estimates relative to the mode point estimates, we used the median point estimates for downstream analyses as the results were more realistic.

Parameter	MRB (Medians)	MRB (Modes)	RRMSE (Medians)	RRMSE (Modes)	RMAE (Medians)	RMAE (Modes)
N1	1.8905	-0.6906	4.889	0.804	0.911	0.890
t	2.1151	-0.7194	5.554	0.805	0.932	0.882
N_{dec}	5.3410	-0.9460	20.894	0.962	0.844	0.995

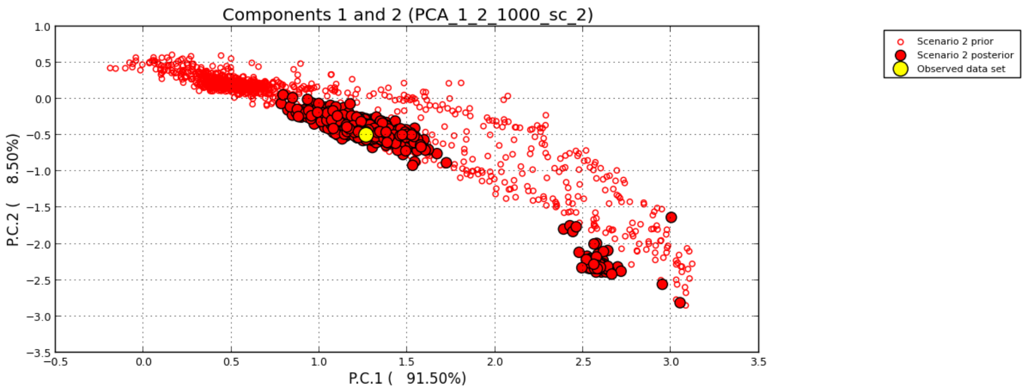

Figure S16: Model checking of the best demographic history scenario (Scenario 2; Fig 3), showing that all posterior sampling of parameters falls within the prior space of the scenario.

Remote sensing and landscape classification details

Table S5: List of remote sensing imagery used for reconstructing historical land-use cover of Singapore. The additional scenes from Feb 2014 and Sep 2014 were used to fill in clouded areas in the June 2013 image.

Scene ID	Date of collection	Spacecraft	Satellite sensor
LT51250591989256BKT00	13 September 1989	Landsat 5	TM
LT51250591997246DKI00	3 September 1997	Landsat 5	TM
LT51250592005124BKT00	4 May 2005	Landsat 5	TM
LC81250592013178LGN01	27 June 2013	Landsat 8	OLI/TIRS
LC81250592014037LGN00	2 Feb 2014	Landsat 8	OLI/TIRS
LC81250592014245LGN00	2 September 2014	Landsat 8	OLI/TIRS

Table S6: Summary of land-use classification categories in Singapore, including inferred habitat value and resistance values for Circuitscape modeling.

Landscape Classification	Description	Habitat Value	Preliminary Resistance	Optimised Resistance
Urban	Built-up areas characterized by high levels of concretization with streetscape vegetation forming low levels of vegetation cover	0.0	60	90
Forest	Wooded areas ranging from primary forests to wasteland habitats largely comprised of tall trees forming some semblance of canopy cover with low to high levels of understorey structure	1.0	1	1
Open Grassland	Areas covered entirely by short grass with little to no tree cover. Grass species are usually *Axonopus compressus* or *Imperata cylindrica*.	0.0	50	50
Bare Ground	Bare, exposed soil with little to no vegetation growing. Often found at sites that have recently been reclaimed or cleared for development.	0.0	100	100
Managed Vegetation	Parkland areas dominated by grassy cover with trees interspersed across the landscape with little to no continuous canopy cover.	0.5	40	50
Inland Water	Shallow water areas including reservoirs, ponds and golf course water hazards.	0.0	100	100
Open Water	Deep water areas in the open sea.	0.0	100	100

Table S7: Resistance model optimization matrix showing the adjusted R^2 values based on a dbMEM analysis using the resistance distance matrix generated by Circuitscape for each combination of urban (U) and managed vegetation (G) resistance values, where U > G.

	G1	G10	G20	G25	G30	G40	G50	G60	G70	G80	G90	G100
0	0.017849736											
0	0.018913564	0.032260003										
5	0.02146266	0.018585581	0.027519648									
0	0.023294363	0.024507499	0.027129823	0.020392289								
0	0.023550216	0.02151023	0.027515521	0.02045581	0.02709894							
0	0.011847449	0.021228613	0.019299849	0.026506301	0.027540559	0.020481142						
0	0.015780106	0.023143029	0.021239218	0.017389268	0.019304113	0.023055162	0.023055012					
0	0.016358954	0.023990601	0.024780974	0.022314746	0.026687265	0.032518683	0.028793461	0.022323425				
0	0.017415185	0.020343348	0.021412519	0.024812029	0.02481614	0.031303117	0.034249874	0.027426186	0.032640665			
0	0.017068384	0.012877676	0.012559012	0.012538532	0.012526637	0.023250072	0.034255372	0.034237061	0.027433062	0.032638744		
00	0.012672204	0.0179595	0.020263848	0.020229558	0.012555893	0.019213072	0.018359577	0.032798331	0.03035696	0.030149519	0.027433961	

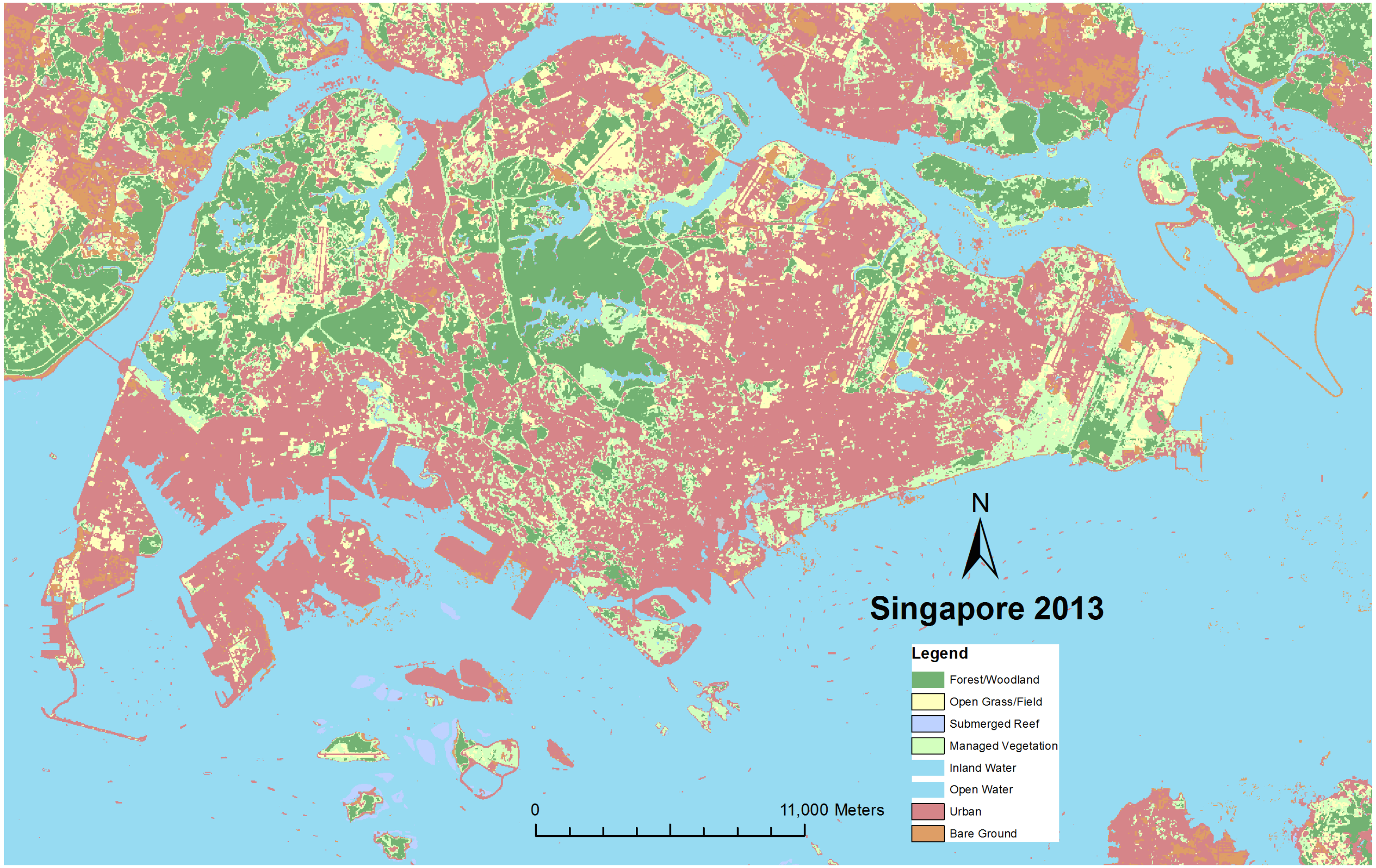

Figure S17: Classified land-use map of Singapore in 2013, showing the distribution of seven habitat types (excluding submerged reefs) defined based on the known habitat preferences of the Striped Tit-Babbler. Land-use was classified using the maximum likelihood supervised classification method in ArcMap v10.0 based on remote sensing imagery from the LandSat 8 OLI/TIRS platform (NASA Landsat Program, 2014d-f).

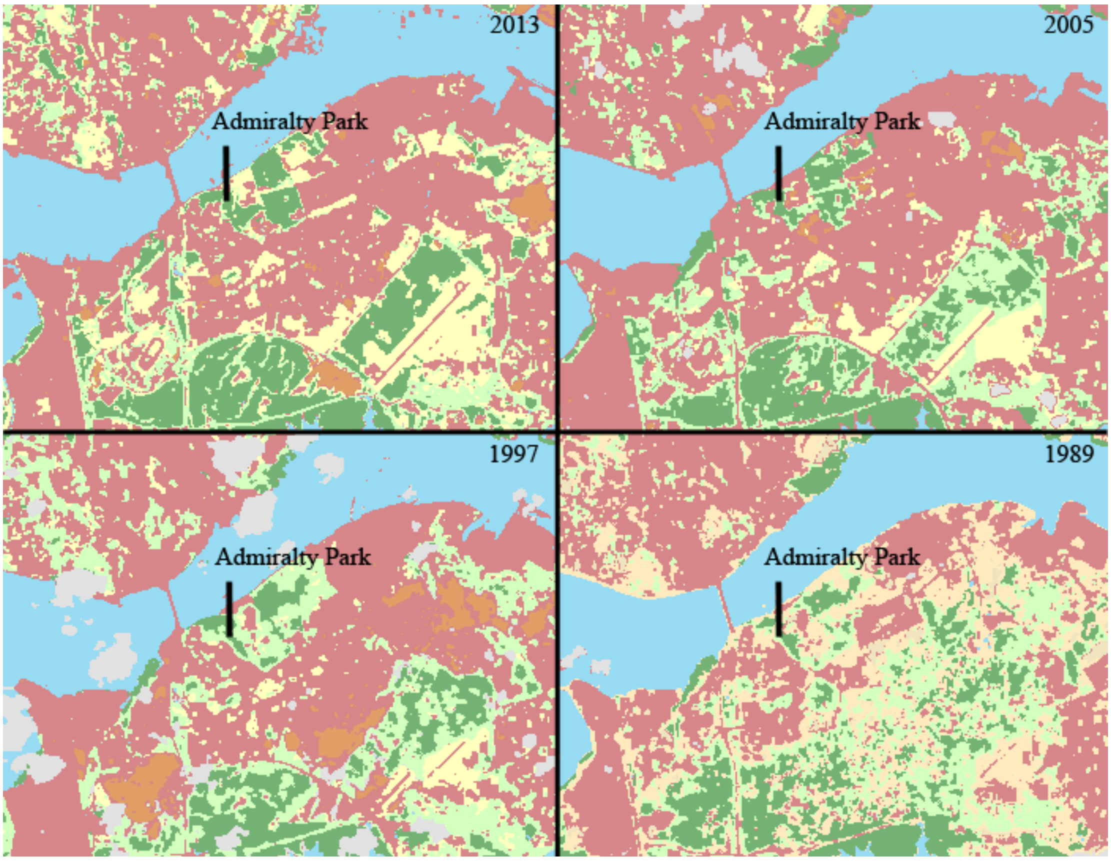

Figure S18: Time-series comparison showing changes in landscape composition around Admiralty Park between 1989 and 2013. The time-series shows the Admiralty Park patch being gradually isolated from the Central Catchment forest, with intervening intermediate habitats cleared for urban development. Land-use was classified using the maximum likelihood supervised classification method in ArcMap v10.0 based on remote sensing imagery from the LandSat 5 TM (NASA Landsat Program, 2014a-c) and LandSat 8 OLI/TIRS (NASA Landsat Program, 2014d-f) platforms.

Landscape Genomic Analyses

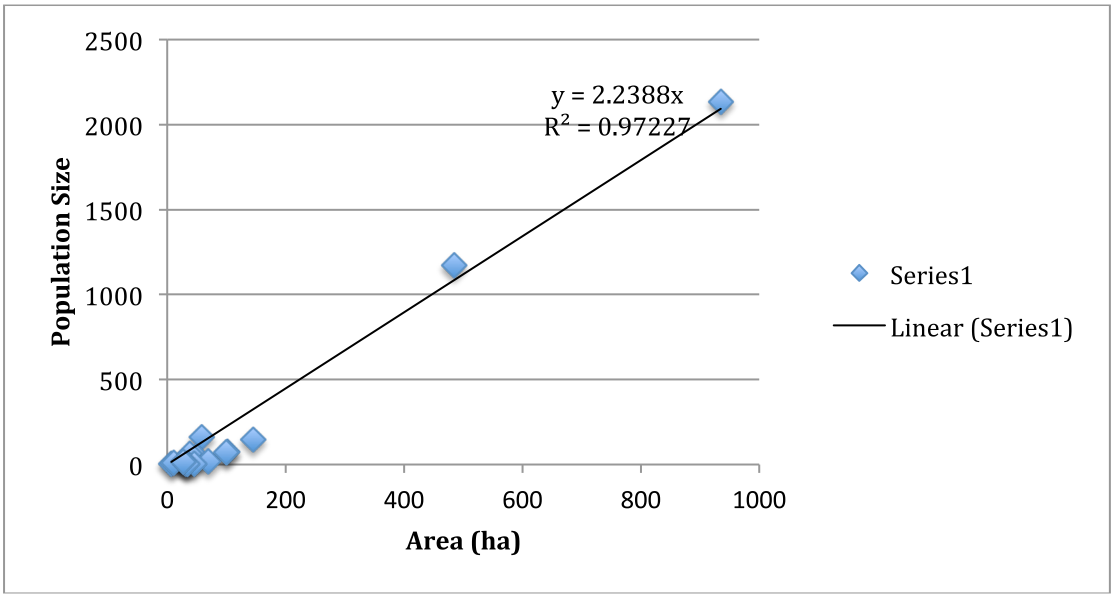

Figure S19: Population size estimation of the Striped Tit-Babbler based on the density estimates and patch areas reported in Castelletta (2001), showing a strong linear correlation between area and population size. Based on this curve, we were able to estimate the approximate population size of the Admiralty Park Striped Tit-Babbler subpopulation at approximately 68.82 individuals.

Table S8: Results of the distance-based Moran's Eigenvector Map (dbMEM) analysis, showing the proportion of variation in genetic distance that is explained by spatial predictors ([abc]), spatial and explained by selected patterns in the model ([a]), spatial and explained by coordinates and not patterns in the model ([c]), spatial and confounded between model and coordinates ([b]), and residual and not explained by spatial predictors ([d]).

model	[abc]	P[abc]	[a]	P[a]	[c]	P[c]	[b]	[d]
Euclidean	0.0275	1.00E-06	0.0249	1.00E-06	0.0150	1.3E-04	-0.0124	0.973
Preliminary Resistance	0.0331	1.00E-06	0.0306	1.00E-06	0.0291	1.00E-06	-0.0268	0.967
Optimised Resistance	0.0344	1.00E-06	0.0318	1.00E-06	0.0291	1.00E-06	-0.0265	0.966

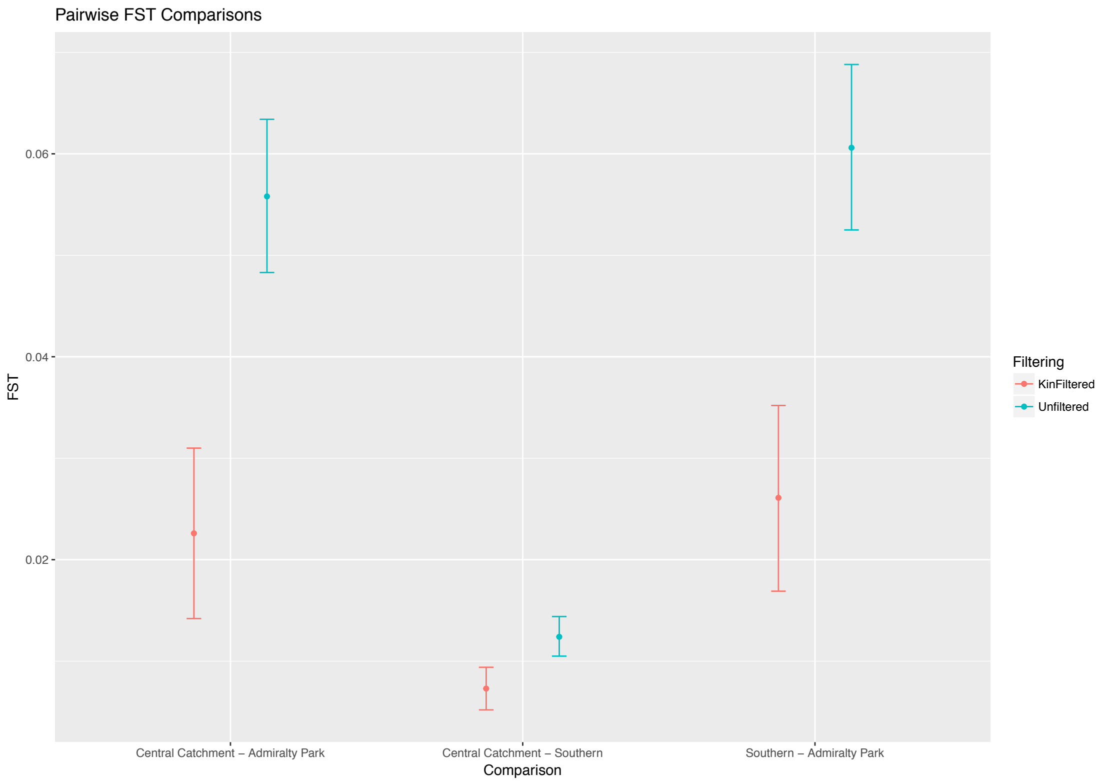

Figure S20: Graphical representation of the filtered and unfiltered Weir and Cockerham's F_{ST} values for each pairwise population comparison, showing low levels of differentiation between the Central Catchment and the Southern subpopulations and relatively higher levels of differentiation between Admiralty Park and every other subpopulation. Values reported in this graph are identical to those reported in Table 3 of the main manuscript.

Literature Cited

Axelsson, E. et al. 2004. Male-Biased Mutation Rate and Divergence in Autosomal, Z-Linked and W-Linked Introns of Chicken and Turkey. - Mol. Biol. Evol. 21: 1538–1547.

Baudouin, L. and Lebrun, P. 2001. AN OPERATIONAL BAYESIAN APPROACH FOR THE IDENTIFICATION OF SEXUALLY REPRODUCED CROSS FERTILIZED POPULATIONS USING MOLECULAR MARKERS. - Acta Hortic.: 81–93.

Beaumont, M. A. 2008. Joint determination of topology, divergence time, and immigration in population trees. - In: Matsumura, S. et al. (eds), Simulation, Genetics, and Human Prehistory. McDonald Institute for Archaeological Research, University of Cambridge, pp. 135–154.

Bolger, A. M. et al. 2014. Trimmomatic: a flexible trimmer for Illumina sequence data. - Bioinformatics 30: 2114–2120.

Bonhomme, V. and Claude, J. 2014. Momocs : Outline Analysis Using R. - J. Stat. Softw. in press.

Bray, J. R. and Curtis, J. T. 1957. An Ordination of the upland forest community of southern Wisconsin.pdf. - Ecol. Monogr. 27: 325–349.

Brown, C. E. 1928. Longevity of Birds in Captivity. - Auk 45: 345–348.

Catchen, J. M. et al. 2011. Stacks: building and genotyping Loci de novo from short-read sequences. - G3 Genes, Genomes, Genet. 1: 171–82.

Catchen, J. et al. 2013. Stacks: an analysis tool set for population genomics. - Mol. Ecol. 22: 3124–40.

Chang, C. C. et al. 2015. Second-generation PLINK: rising to the challenge of larger and richer datasets. - Gigascience 4: 1–16.

Cockburn, A. 2006. Prevalence of different modes of parental care in birds. - Proc. R. Soc. B Biol. Sci. 273: 1375–1383.

Corlett, R. T. 1992. The ecological transformation of Singapore , 1819-1990. - J. Biogeogr. 19: 411–420.

Corlett, R. T. 1997. The Vegetation in the Nature Reserves of Singapore. - Gard. Bull. Singapore 49: 147–159.

Cornuet, J.-M. et al. 2014. DIYABC v2.0: a software to make approximate Bayesian computation inferences about population history using single nucleotide polymorphism, DNA sequence and microsatellite data. - Bioinformatics 30: 1187–1189.

Cushman, S. A. et al. 2006. Gene flow in complex landscapes: testing multiple hypotheses with causal modeling. - Am. Nat. 168: 486–99.

Do, C. et al. 2014. <scp>NeEstimator</scp> v2: re-implementation of software for the estimation of contemporary effective population size (N_e) from genetic data. - Mol. Ecol. Resour. 14: 209–214.

Doyle, R. W. 2014. Inbreeding and disease in tropical shrimp aquaculture: a reappraisal and caution. - Aquac. Res. in press.

Dray, S. and Dufour, A. B. 2007. The ade4 package: implementing the duality diagram for ecologists. - J. Stat. Softw. 22: 1–20.

Fagundes, N. J. R. et al. 2007. Statistical evaluation of alternative models of human evolution. - Proc. Natl. Acad. Sci. 104: 17614–17619.

Fogden, M. P. L. 1972. THE SEASONALITY AND POPULATION DYNAMICS OF EQUATORIAL FOREST BIRDS EST SARAWAK. - Ibis (Lond. 1859). 114: 307–343.

Foll, M. and Gaggiotti, O. 2008. A Genome-Scan Method to Identify Selected Loci Appropriate for Both Dominant and Codominant Markers: A Bayesian Perspective. - Genetics 180: 977–993.

Galpern, P. et al. 2014. MEMGENE: Spatial pattern detection in genetic distance data (O Pybus, Ed.). - Methods Ecol. Evol. 5: 1116–1120.

Gelang, M. et al. 2009. Phylogeny of babblers (Aves, Passeriformes): major lineages, family limits and classification. - Zool. Scr. 38: 225–236.

Gnerre, S. et al. 2011. High-quality draft assemblies of mammalian genomes from massively parallel sequence data. - Proc. Natl. Acad. Sci. U. S. A. 108: 1513–1518.

Goslee, S. C. and Urban, D. L. 2007. The ecodist Package for Dissimilarity-based Analysis of

Ecological Data. - J. Stat. Softw. 22: 1–19.
Keenan, K. et al. 2013. diveRsity : An R package for the estimation and exploration of population genetics parameters and their associated errors (RB O'Hara, Ed.). - Methods Ecol. Evol. 4: 782–788.
Khoonwongsa, J. 2011. Post Fledging Survival and Juvenile Dispersal in Abbott's Babbler (Malacocincla abbotti) in Khao Yai National Park.
Kimura, M. and Crow, J. F. 1964. The number of alleles that can be maintained in a finite population. - Genetics 49: 725–738.
Landguth, E. L. and Cushman, S. A. 2010. cdpop: A spatially explicit cost distance population genetics program. - Mol. Ecol. Resour. 10: 156–161.
Langmead, B. and Salzberg, S. L. 2012. Fast gapped-read alignment with Bowtie 2. - Nat. Methods 9: 357–359.
Legendre, L. and Legendre, P. 1998. Numerical Ecology. - Elsevier.
Legendre, P. et al. 2015. Should the Mantel test be used in spatial analysis? (P Peres-Neto, Ed.). - Methods Ecol. Evol. 6: 1239–1247.
Li, H. 2011. A statistical framework for SNP calling, mutation discovery, association mapping and population genetical parameter estimation from sequencing data. - Bioinformatics 27: 2987–2993.
Li, H. and Durbin, R. 2009. Fast and accurate short read alignment with Burrows-Wheeler transform. - Bioinformatics 25: 1754–1760.
Li, R. et al. 2010. De novo assembly of human genomes with massively parallel short read sequencing. - Genome Res. 20: 265–272.
Mastretta-Yanes, A. et al. 2014. Restriction site-associated DNA sequencing, genotyping error estimation and de novo assembly optimization for population genetic inference. - Mol. Ecol. Resour.: 28–41.
McRae, B. H. et al. 2008. Using Circuit Theory to Model Connectivity In Ecology, Evolution, and Conservation. - Ecology 89: 2712–2724.
McRae, B. H. et al. 2013. Circuitscape 4 User Guide.
McRae, B. H. et al. 2014. Gnarly Landscape Utilities: Resistance and Habitat Calculator User Guide.
Meirmans, P. G. and Van Tienderen, P. H. 2004. GENOTYPE and GENODIVE: Two programs for the analysis of genetic diversity of asexual organisms. - Mol. Ecol. Notes 4: 792–794.
NASA Landsat Program. 2014a. Landsat TM scene LT51250591989256BKT00. L1TP. Sioux Falls. 13/09/1989.
NASA Landsat Program. 2014b. Landsat TM scene LT51250591997246DKI00. L1TP. Sioux Falls. 03/09/1997.
NASA Landsat Program. 2014c. Landsat TM scene LT51250592005124BKT00. L1TP. Sioux Falls. 04/05/2005.
NASA Landsat Program. 2014d. Landsat OLI/TIRS scene LC81250592013178LGN01. L1TP. Sioux Falls. 27/06/2013.
NASA Landsat Program. 2014e. Landsat OLI/TIRS scene LC81250592014037LGN00. L1TP. Sioux Falls. 02/02/2014.
NASA Landsat Program. 2014f. Landsat OLI/TIRS scene LC81250592014245LGN00. L1TP. Sioux Falls. 02/09/2014.
Nei, M. 1987. Molecular Evolutionary Genetics. - Columbia University Press.
O'Dempsey, T. 2014. Singapore's Changing Landscape Since c. 1800. - In: Barnard, T. P. (ed), Nature Contained: Environmental Histories of Singapore. NUS Press, pp. 328.
Olsen, R.-A. et al. 2015. De novo assembly of Dekkera bruxellensis: a multi technology approach using short and long-read sequencing and optical mapping. - Gigascience 4: 56.
Paetkau, D. et al. 2004. Genetic assignment methods for the direct, real-time estimation of migration rate: a simulation-based exploration of accuracy and power. - Mol. Ecol. 13: 55–65.
Peakall, R. and Smouse, P. E. 2012. GenALEx 6.5: Genetic analysis in Excel. Population genetic software for teaching and research-an update. - Bioinformatics 28: 2537–2539.
Peng, Y. 2006. Population genetic structure of Steere's liocichla (Liocochla steerii) in Taiwan.

Peterson, B. K. et al. 2012. Double digest RADseq: An inexpensive method for de novo SNP discovery and genotyping in model and non-model species. - PLoS One in press.
Pew, J. et al. 2015. related: an R package for analysing pairwise relatedness from codominant molecular markers. - Mol. Ecol. Resour. 15: 557–561.
Piry, S. et al. 2004. GENECLASS2: A Software for Genetic Assignment and First-Generation Migrant Detection. - J. Hered. 95: 536–539.
Queller, D. C. and Goodnight, K. F. 1989. Estimating Relatedness Using Genetic Markers. - Evolution (N. Y). 43: 258–275.
Schmidt, T. L. et al. 2017. Fine-scale landscape genomics of Aedes aegypti reveals loss of Wolbachia transinfection, dispersal barrier and potential for occasional long distance movement. - bioRxiv in press.
Seutin, G. et al. 1991. Preservation of avian blood and tissue samples for DNA analyses. - Can. J. Zool. 69: 82–90.
Simpson, J. T. et al. 2009. ABySS: A parallel assembler for short read sequence data. - Genome Res. 19: 1117–1123.
Tay, Y. C. et al. 2016. Beyond the Coral Triangle: high genetic diversity and near panmixia in Singapore's populations of the broadcast spawning sea star Protoreaster nodosus. - R. Soc. Open Sci. 3: 160253.
Tin, M. M. Y. et al. 2015. Degenerate adaptor sequences for detecting PCR duplicates in reduced representation sequencing data improve genotype calling accuracy. - Mol. Ecol. Resour. 15: 329–336.
Wang, J. 2007. Triadic IBD coefficients and applications to estimating pairwise relatedness. - Genet. Res. 89: 135–153.
Wang, J. 2011. Coancestry: A program for simulating, estimating and analysing relatedness and inbreeding coefficients. - Mol. Ecol. Resour. 11: 141–145.
Wang, L. K. and Hails, C. J. 2007. An Annotated Checklist of the Birds of Singapore. - Raffles Bull. Zool. Supplement: 1–179.
Waples, R. S. and Anderson, E. C. 2017. Purging putative siblings from population genetic data sets: a cautionary view. - Mol. Ecol. 26: 1211–1224.
Weir, B. S. and Cockerham, C. C. 1984. Estimating F-Statistics for the Analysis of Population Structure. - Evolution (N. Y). 38: 1358.
Wells, D. 2007. Birds of the Thai-Malay Peninsula: Passerines: Volume 2. - Christopher Helm Publishers Ltd.
Willing, E. M. et al. 2012. Estimates of genetic differentiation measured by fst do not necessarily require large sample sizes when using many snp markers. - PLoS One 7: 1–7.
Yee, A. T. K. et al. 2011. The vegetation of Singapore — an updated map. - Gard. Bull. Singapore 63: 205–212.
Yong, D. L. 2009. Persistence of Babbler (Timaliidae) Communities in Singapore Forests. - Nat. Singapore 2: 365–371.
